# Complete tag loss in capture–recapture studies affects abundance estimates: An elephant seal case study

**DOI:** 10.1002/ece3.6052

**Published:** 2020-01-30

**Authors:** Emily Malcolm‐White, Clive R. McMahon, Laura L. E. Cowen

**Affiliations:** ^1^ Mathematics Middlebury College Middlebury VT USA; ^2^ Sydney Institute for Marine Science Mosman NSW Australia; ^3^ Mathematics and Statistics University of Victoria Victoria BC Canada

**Keywords:** abundance, capture–mark–recapture, complete tag loss, demography, double tagging, elephant seal, Jolly‐Seber, recycled individual

## Abstract

1. In capture–recapture studies, recycled individuals occur when individuals lose all of their tags and are recaptured as though they were new individuals. Typically, the effect of these recycled individuals is assumed negligible.
2. Through a simulation‐based study of double‐tagging experiments, we examined the effect of recycled individuals on parameter estimates in the Jolly–Seber model with tag loss (Cowen & Schwarz, 2006). We validated the simulation framework using long‐term census data of elephant seals.
3. Including recycled individuals did not affect estimates of capture, survival, and tag‐retention probabilities. However, with low tag‐retention rates, high capture rates, and high survival rates, recycled individuals produced overestimates of population size. For the elephant seal case study, we found population size estimates to be between 8% and 53% larger when recycled individuals were ignored.
4. Ignoring the effects of recycled individuals can cause large biases in population size estimates. These results are particularly noticeable in longer studies.

## Graphical abstract

In capture‐recapture studies, recycled individuals occur when individuals lose all of their tags and are recaptured as though they were new individuals. Ignoring the effects of recycled individuals can cause large biases in population size estimates.

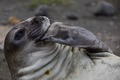

## 1 INTRODUCTION

Mark–recapture studies utilize statistical techniques to estimate population parameters. Over *k* sample times, individuals are captured, tagged with unique tags, released and potentially recaptured at subsequent sampling times. The Jolly–Seber model (Jolly, 1965; Seber, 1965) is used to model open populations since it can estimate parameters of interest such as population size and survival rates (Pollock, Nichols, Brownie, & Hines, 1990). An important assumption of this model is that individuals never lose their tags. However, when this assumption is violated, serious bias can occur in the parameter and variance estimates (Arnason & Mills, 1981). Double tagging, the placement of two tags on an individual, can be used to estimate tag‐retention rates. Double‐tagging studies have been used for a wide variety of species (for example cod: Björnsson, Karlsson, Thorsteinsson, & Solmundsson, 2011; lobsters: Xu, Cowen, Garder, 2014; sea turtles: Bjorndal, Bolten, Lagueux, & Chaves, 1996; elephant seals: Pistorius, Bester, Kirkman, & Boveng, 2000; black bears: Diefenbach & Alt, 1998) to investigate probabilities of tag loss or tag shedding rates. Often, a mixture of single‐ and double‐tagged individuals is used for practical purposes. Cowen and Schwarz (2006) incorporated tag loss by developing the Jolly‐Seber tag loss (JSTL) model for experiments where some fraction of individuals are double‐tagged. This model was further extended to account for heterogeneity in capture between groups (Xu et al., 2014). In the simplest form of the JSTL model, it is assumed that every individual present in the population at sample time *k* has capture, survival, and tag‐retention probabilities that are homogeneous for all individuals in the population across all sampling occasions. However, these assumptions are rarely met and can induce significant bias in the parameter estimates (Schwarz, Hindell, McMahon, & Costa, 2012).

Occasionally in mark–recapture experiments, previously captured individuals lose all of their tags (complete tag loss). These individuals are either recognized upon recapture (for example, through scarring or fin clipping), and not retagged, or if unrecognized, these individuals would be tagged again and treated as “new” individuals. Individuals who lose both tags and are recaptured and retagged are known as recycled individuals. For example, an individual with the tag history {11 01 00} over three sampling occasions was double‐tagged at sample time 1, lost a tag between times 1 and 2, and may have lost its last tag between sample times 2 and 3 and then have been recaptured at sample time 3 resulting in a new individual with tag history {00 00 11}. If the rate of tag loss is small, bias in the population estimate will also be small for the Peterson estimators (Seber & Felton, 1965). Typically in the Jolly‐Seber and JSTL models, the effect of recycled individuals is assumed to be negligible. However, in situations where tag retention is low and survival and recapture probabilities are high, it is suspected that recycled individuals will bias population size estimates upwards. The motivation for this study was to investigate the effect of recycled individuals on parameter estimates in the JSTL model through a simulation study and determine under which conditions researchers need to be concerned. This study is important as the assumption that the effect is negligible has not been fully tested and quantified, and most studies that rely on marking individuals typically experience tag loss. Thus, there is a need to account for recycled individuals given the desire for accurate and robust estimates for management and conservation purposes.

**Figure 1:**
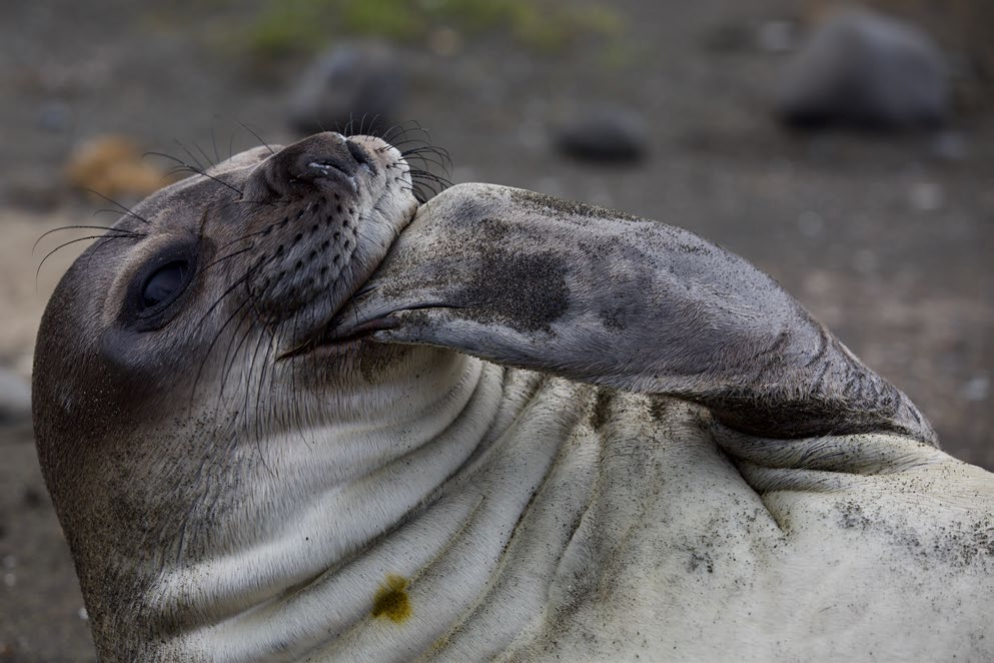
Southern elephant seal (*Mirounga leonina*) at Macquarie Island, Australia

In order to determine whether the simulation framework provided a reasonable approximation to the real world, we analyzed the effects of recycled individuals in long‐term census data of southern elephant seals (Figure 1).

## 2 MATERIALS AND METHODS

### 2.1 The Jolly–Seber Model with Tag Loss

Full development of the JSTL model is given by (Cowen & Schwarz, 2006). Data are typically in the form of tag histories for each individual in the study. Let *ω_ijd_* denote the entries of the tag history for individual *i* at time *j*, tag *d*; *i* = 1,…,*n*_obs_, *j* = 1, …, *k*, *d* = 1,2; with *ω_ijd_* equaling 1 or 0 if the individual was seen with tag *d* or not. Capture histories *ω* ^*^ used in most capture–recapture studies can be easily obtained from the tag histories *ω* and are a series of 1's and 0's depicting whether or not an individual was captured at each sample time. We focus on the case with constant parameters as used in our simulation study. Let *ϕ* denote the survival probability, *p* the capture probability, *λ* the tag‐retention probability, and *b_t_* the entry probability for time *t*. Ψ and *χ* are recursive functions of parameters that deal with an individual's history up to the first time seen (*f_i_*) and after the last time seen (*l_i_*) with *n_t_* tags, respectively.

The JSTL model is developed under the idea of a super‐population (the number of individuals that will enter the population at some point during the study; Schwarz & Arnason, 1996), and this allows the likelihood to be formulated into two parts: (a) a model for the observed number of unique tag histories (*n*_obs_) given the super‐population size (*N*) and (b) a model for the recaptures (in the form of capture history frequencies $n_{\omega_{i}}$) given the observed number of unique tag histories (*n*_obs_). The full likelihood is given by the product of these components as follows:

$$\begin{array}{c} L=\left(\begin{array}{c} N\\ n_{\text{obs}} \end{array}\right){\left\{\sum_{j=0}^{k-1}b_{j}\left(1-p\right)\chi_{\left(0,j+1,0\right)}\right\}}^{\left(N-n_{\text{obs}}\right)}{\left\{1-\sum_{j=0}^{k-1}b_{j}\left(1-p\right)\chi_{\left(0,j+1,0\right)}\right\}}^{n_{\text{obs}}}\times \\ \;\left(\begin{array}{c} n_{\text{obs}}\\ n_{\omega_{1}},n_{\omega_{2}},\ldots ,n_{\omega_{m}} \end{array}\right)\prod_{i=1}^{m}{\left[\psi_{f_{i}}T_{d}\left\{\prod_{j=f_{i}}^{l_{i}}p^{\omega_{\mathrm{ij}}^{*}}\right\}\left\{\prod_{j=f_{i}}^{l_{i}-1}\phi \right\}\times \prod_{d=1}^{2}\left\{\left(\prod_{j=f_{i}}^{l_{\mathrm{id}}-1}\lambda \right){\left(1-\prod_{j=l_{\mathrm{id}}}^{q_{\mathrm{id}}-1}\lambda \right)}^{I\left(l_{\mathrm{id}}\neq l_{i}\right)}\right\}\times \chi_{f_{i},l_{i},nt_{l_{i}}}\right]}^{n_{\omega_{i}}}\times \\ \;{\left\{1-\sum_{j=0}^{k-1}b_{j}\left(1-p\right)\chi_{\left(0,j+1,0\right)}\right\}}^{-n_{\text{obs}}} \end{array}$$

where *T_d_* is the probability of being double‐tagged, *l_id_* is the last sample time where tag *d* was present, and *nt_j_* is the number of tags on individual *i* at time *j*. A table of notation is provided in Appendix A with further details on the Ψ and *χ* functions.

Assumptions of the JSTL model (under constant *ϕ, p*, and *λ* parameters) are similar to the Jolly–Seber model (Schwarz & Arnason, 1996) including all individuals have equal entry (birth or immigration) probabilities but entry probabilities can vary between sample times, capture probabilities are the same for all individuals at all sample times, all individuals (marked and unmarked) have equal survival probabilities between all sample times, the sampling period is relatively short compared with the interval between sampling times, and there is independence across all individuals. The incorporation of tag loss into the model comes with the additional assumption that all marked individuals have equal tag‐retention probabilities between all sample times and for double‐tagged individuals, and tag loss is independent between tags. Finally, the JSTL model assumes that the effect of recycled individuals is negligible and it is this assumption that we explore.

Many different models can be specified for the JSTL model where parameters are homogeneous or heterogeneous with respect to time (Cowen & Schwarz, 2006) or group (Xu et al., 2014).

### 2.2 Likelihood and estimation

Maximum likelihood parameter estimates are found using a Newton–Raphson type method. Estimated standard errors are computed using the delta theorem. Models were implemented using R software (R Core Team, 2014). Code from this study are included in this published article (and its Appendix S1).

### 2.3 Experimental design

To study the effect of recycled individuals on parameter estimates of this model, we conducted a simulation study. Data sets varied in super‐population size, parameter values, and percent double‐tagged. We generated data for the JSTL model with constant survival, capture, and tag‐retention probabilities for a double‐tagging experiment. Super‐population sizes of 1,000 and 100,000 were considered in order to study the effect of population size. For the super‐population size of 100,000, experiments with ten sample times were considered. For the super‐population size of 1,000, we considered experiments with five, seven, and ten sample times in order to determine the effect of the study length. For each population size, we tested different proportions of double‐tagged versus single‐tagged individuals (0.5 and 1). Survival, capture, and tag‐retention probability parameters were varied in a 3^3^ experimental design with low (0.2), medium (0.5), and high (0.9) values for all parameters. The entry rates were fixed to be 1/*k* at each of the sampling times.

We considered the set of parameter values to be reasonable values that might be encountered in practice and also produce informative capture–recapture scenarios. Tag‐retention rates can vary by species, age of the tag, tag type, tag location, behaviour, season, and individual quality (size of an animal for example in seals). For example, tag‐retention rates have ranged from 13% (Fogarty, Borden, & Russell, 1980) to 95% (Gonzalez‐Vicente, Diaz, Mallol, & Goni, 2012) in lobsters. Other studies report tag‐retention rates of 65% in male elephant seals (Pistorius et al., 2000) and 88% in Adelie penguins (Ainley & DeMaster, 1980). Mean retention of visible implant tags has been recorded as 32% in small rockpool fish (Griffiths, 2002). Turtles in particular experience high tag loss rates. For example, Bellini, Godfrey, and Sanches (2001) reports the probability of tag loss in hawksbill turtles as 0.57 and Bjorndal et al. (1996) observed the probability of tag loss in green nesting turtles to be as high as 0.38. Thus, we chose a wide range of tag loss parameter values to try to capture the diversity among published tag loss rates.

### 2.4 Simulation of data

For all of the parameter combinations of super‐population size (*N* = 1,000, 100,000), fraction double‐tagged (0.5, 1), survival probability (*ϕ* = 0.2, 0.5, 0.9), capture probability (*p* = 0.2, 0.5, 0.9), and tag‐retention probability (*λ* = 0.2, 0.5, 0.9), we generated 100 data sets where the simulated data met all the assumptions of the model.

For each individual, we simulated a capture history using the following algorithm:

1. Determine when the individual enters the population utilizing the entry probabilities.
2. For each sample time after entry (until death or first capture), determine if the individual survives to that sample time (with probability *ϕ*). If they are still alive, determine if they are first captured (with probability *p*). If they are captured, determine whether they are single‐ or double‐tagged.
3. For each sample time after first capture (until death, loss of all tags or the end of the study), determine if the individual survives to that sample time (with probability *ϕ*). Then, if they are still alive, determine if they lose any of their tags (with probability 1–*λ*). If they still have at least one of their tags, determine if they are recaptured (with probability *p*). If they have lost all of their tags, consider them as a new individual entering the population at this sample time.

By keeping track of all the recycled individuals, this algorithm provides us with two data sets: one that includes the recycled individuals (assumes individuals, who have complete tag loss, are tagged again upon recapture and treated as new individuals) and one that excludes recycled individuals (assumes that individuals, who have complete tag loss, can be recognized upon recapture and are not retagged). The JSTL model was fit to the 100 simulated data sets twice (once including and once excluding recycled individuals). We assumed that any difference between the two analyses was due entirely to the recycled individuals. All data generated during this study are included in this published article (and its Appendix S1).

### 2.5 Evaluation criteria

To evaluate the resulting parameter estimates from each of the simulations, we looked at several criteria including: average parameter estimate, relative bias of the estimates, the average standard error of the parameter estimates, the standard deviation of the parameter estimates, and root mean squared error (RMSE) of the parameter estimates.

Let ${\hat{\theta }}_{i}$'s be the parameter estimates from each of the 100 simulations and *θ* the true parameter value, we calculated:

- the mean parameter estimate as $\bar{\hat{\theta }}=\frac{1}{100}\sum_{i=1}^{100}{\hat{\theta }}_{i}$
- average standard error of the parameter estimate as $SE\left(\hat{\theta }\right)=\frac{1}{100}\sum_{i=1}^{100}SE({\hat{\theta }}_{i})$.
- the standard deviation of the parameter estimates as $SD\left(\hat{\theta }\right)=\sqrt{\frac{1}{99}\sum_{i=1}^{100}{\left({\hat{\theta }}_{i}-\bar{\hat{\theta }}\right)}^{2}}$.
- the RMSE of the parameter estimates as $\text{RMSE}=\sqrt{\frac{1}{100}\sum_{i=1}^{100}{\left({\hat{\theta }}_{i}-\bar{\hat{\theta }}\right)}^{2}}$.

We compared the average parameter estimates to the true parameter values using relative bias. We calculated the relative bias of the estimators as $(\bar{\hat{\theta }}-\theta )/\theta$. We also compared the relative bias from the analysis with the recycled individuals to the relative bias from the analysis without the recycled individuals. We calculated the difference in the two relative biases and consider this to be the relative bias that was contributed entirely by the recycled individuals being tagged as “new” individuals.

## 3 SIMULATION RESULTS

**Figure 2:**
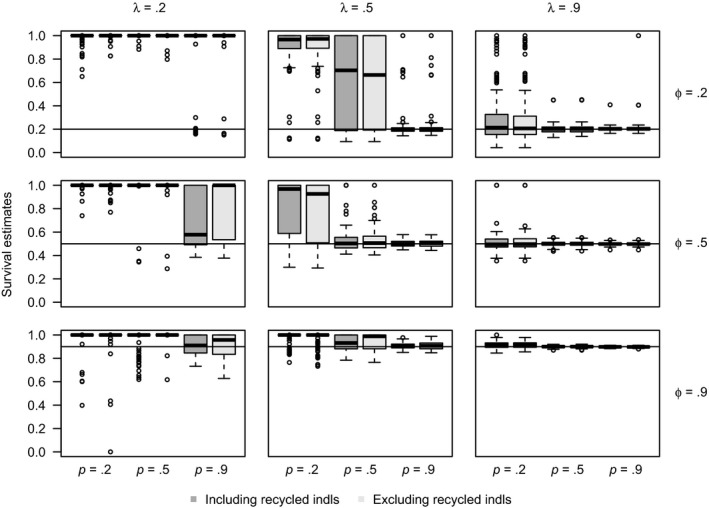
Survival probability estimates for simulated data with super‐population size *N* = 1,000 with 100% double tagging for different tag‐retention probabilities (*λ* = 0.2, 0.5, 0.9), survival probabilities (*ϕ* = 0.2, 0.5, 0.9), and different capture probabilities (*p* = 0.2, 0.5, 0.9) using the JSTL model from a ten‐sample‐time study. Box plots of the estimates of *ϕ* for the model analyzed including and excluding the recycled individuals are provided. The black line indicates the true value of *ϕ* used to simulate the data for each model

The survival estimates are biased for some parameter combinations of survival, capture, and tag‐retention probabilities. As an example, box plots of survival estimates for data with super‐population size *N* = 1,000 and 100% double tagging are provided (Figure 2). Box plots of survival estimates for other super‐population sizes and double‐tagging rates are provided in the Appendix S1 (Figures A1–A6). Although there is bias in the survival estimates for several of the parameter combinations, the bias is similar between the analysis including and excluding the recycled individuals for both super‐population sizes (*N* = 1,000 and 100,000) and for both double‐tagging rates (*T*_2_ = 0.5, 1). In fact, the differences in relative bias due to recycled individuals for the parameters *ϕ*, *p,* and *λ* is small (<0.01) for all 108 parameter combinations considered. In general, the *SE*, *SD*, and RMSE of the estimates of *ϕ*, *p*, and *λ* are similar for both the analysis including and excluding recycled individuals for the parameter combinations considered. It seems that the treatment of recycled individuals has little effect, if any, on the accuracy of the JSTL estimators for survival, capture, and tag‐retention probabilities. Box plots of capture and tag‐retention estimates for all models can also be found in the Appendix S1 (Figures A7–A17).

There is slightly more bias due to recycled individuals for parameter combinations where the probability of double tagging (*T*_2_) was only 0.5, compared with the parameter combinations where all individuals were double‐tagged. As an example, relative bias of the parameters are presented for the parameter combination where *ϕ* = 0.9, *p* = 0.9, and *λ* = 0.2 for both the analysis including and excluding recycled individuals for varying population size and double‐tagging probabilities (Table 1).

**Table 1:** The mean relative bias of the parameters from the model analyzed including (*R*) and excluding (*R′*) the recycled individuals for data with high survival probability (*ϕ* = 0.9), high capture probability (*p* = 0.9), and low tag retention (*λ* = 0.2) for different super‐populations sizes (*N* = 1,000, 100,000) and different proportion double‐tagged (*T*_2_ = 0.5, 1) using the JSTL model from a ten‐sample‐time study

	*N* = 1,000				*N* = 100,000			
	*T*_2_ = 1		*T*_2_ = 0.5		*T*_2_ = 1		*T*_2_ = 0.5	
	*R*	*R′*	*R*	*R′*	*R*	*R′*	*R*	*R′*
*ϕ*	0.00	0.00	0.06	0.05	0.00	0.00	0.11	0.10
*p*	0.00	0.00	0.00	0.00	0.00	0.00	0.00	0.00
*λ*	0.00	0.00	0.00	0.00	0.00	0.00	−0.09	−0.08
*N*	1.98	0.00	2.13	0.00	1.98	0.00	2.12	0.00

**Figure 3:**
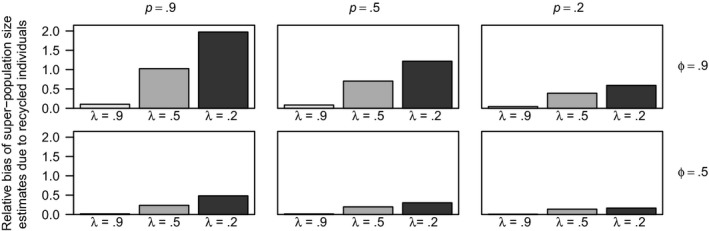
The difference in mean relative bias of the super‐population estimate $\left(\hat{N}\right)$ between the model analyzed including and excluding the recycled individuals for data with super‐population size *N* = 100,000 with 100% double tagging for different tag‐retention probabilities (*λ* = 0.2, 0.5, 0.9), survival probabilities (*ϕ* = 0.5, 0.9), and capture probabilities (*p* = 0.2, 0.5, 0.9) using the JSTL model from a ten‐sample‐time study

The estimate of super‐population size $\left(\hat{N}\right)$ is computed as $\hat{N}=n_{\text{obs}}/\left(1-{\hat{P}}_{0}\right)$, where ${\hat{P}}_{0}$ is the estimated probability of never being seen. In the scenarios where many recycled individuals were recaptured and considered as “new” individuals (included), the number of observed individuals, *n*_obs_, is larger than it should be, and thus, $\hat{N}$ is biased upwards. By recognizing recycled individuals upon recapture, this bias can be corrected. The relative bias in the super‐population size $\left(\hat{N}\right)$ due to recycled individuals is highest in the scenario with high survival rates (*ϕ* = 0.9), high capture rates (*p* = 0.9), and low tag‐retention rates (*λ* = 0.2), as predicted (Figure 3, Table 1). The relative bias is small for all scenarios where tag retention was high, but relative bias increases as tag retention decreases. The relative bias in $\hat{N}$ decreases as capture probability decreases, but recycled individuals appear to still have some effect on the estimates even when capture probabilities are low (*p* = 0.2). The relative bias in $\hat{N}$ is high for scenarios where survival probability is high and decreases as survival probability decreases. In all scenarios where survival probability is low (*ϕ* = 0.2), individuals are unlikely to survive long enough to be able to be tagged, lose tag(s) and be recaptured as “new” individuals. When survival probability is low, the relative bias due to the recycled individuals is small (<0.15) and hence not shown in Figure 3. *SE*, *SD*, and RMSE of $\hat{N}$ varies, but remains similar between the analyses including and excluding recycled individuals, across all scenarios.

**Figure 4:**
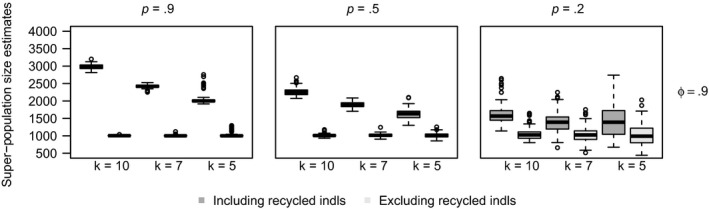
Box plots of the estimates of *N* for the model analyzed including and excluding the recycled individuals for data with super‐population size *N* = 1,000 with 100% double tagging for different capture probabilities (*p* = 0.2, 0.5, 0.9), and constant survival (*ϕ* = 0.9) and tag‐retention (*λ* = 0.2) probabilities using the JSTL model from experiments with *k* = 10, 7, and 5 sample times

There is more bias in $\hat{N}$ due to recycled individuals in longer experiments (Figure 4). With a larger number of sampling occasions, there is more time for individuals to be captured and tagged, lose their tags, and survive to be recaptured (be recycled). In shorter studies, there are fewer numbers of recycled individuals and thus the bias in $\hat{N}$ due to recycled individuals is lower although not unnoticeable in the worst case scenarios (low tag retention, high survival, and high capture probabilities). Box plots of super‐population size (*N*) for all scenarios are available in the Appendix S1 (Figures A19–A24).

**Figure 5:**
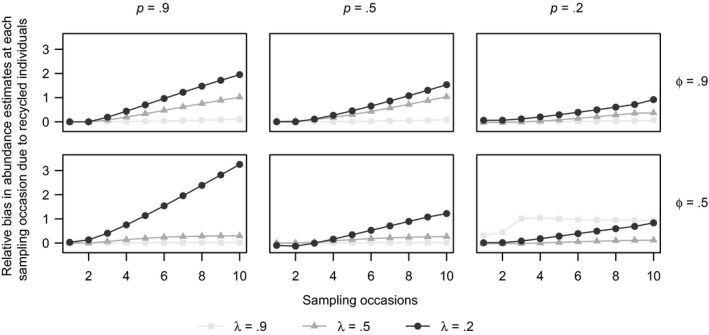
The difference in mean relative bias of the abundance estimates at each sample time $\left({\hat{N}}_{j}\right)$ between the model analyzed including and excluding the recycled individuals for data with super‐population size *N* = 100,000 with 100% double tagging for different tag‐retention probabilities (*λ* = 0.2, 0.5, 0.9), survival probabilities (*ϕ* = 0.5 and 0.9), and different capture probabilities (*p* = 0.2, 0.5, 0.9) using the JSTL model from a ten‐sample‐time study. Note that lines are added between the points to emphasize the difference in values; no models were fit to generate these lines

In general, the bias due to recycled individuals in the ${\hat{N}}_{j}$'s follows a similar pattern to the bias due to recycled individuals in$\hat{N}$, with relative bias in the ${\hat{N}}_{j}$'s increasing as tag retention decreases, survival increases, and capture probability increases (Figure 5). For all scenarios, the relative bias in the estimates of abundance at each sample time *j* is larger later in the study. Since the estimates of the population sizes at each time *j* are computed iteratively as ${\hat{N}}_{j+1}=\hat{\phi }\left({\hat{N}}_{j}\right)+{\hat{b}}_{j}\left(\hat{N}\right)$, any bias in the earlier abundance estimates is magnified in the later sampling occasions abundance estimates. The scenario with *ϕ* = 0.5, *p* = 0.9 and *λ* = 0.2 appears to have very high relative bias in the abundance estimates in later sampling occasions (>3 for ${\hat{N}}_{10}$), which is caused by a combination of more upward bias in the survival probability estimates for the analysis including recycled individuals (Figures A1–A6) as well as upward bias in the super‐population size estimates. Plots of the mean abundance estimates for all scenarios are available in the Appendix S1 (Figures A25–A42).

## 4 CASE STUDY: ELEPHANT SEALS

To validate the simulation framework, we analyzed seven years of data from a long‐term mark‐recapture study of elephant seals on Macquarie Island, Australia between 1993 and 2000. Elephant seal pups were marked with two tags in the inter‐digital webbing of their hind flippers and were given a permanent hot‐iron branding with a unique identifier on their flank (McMahon, Burton, Hoff, Woods, & Bradshaw, 2009). This permanent branding allowed for individual elephant seals to be identified even if they lost both tags. Thus, recycled individuals could be easily identified.

We considered two analyses of the data:

1. We assumed that recycled individuals could not be recognized upon recapture (ignoring branding) and were retagged as if they were new individuals. *Recycled individuals are included.* This scenario simulates analysis ignoring the effects of recycled individuals.
2. Recycled individuals were recognized upon recapture (by branding) and were retagged with new tags identical to their lost tags. Thus, *recycled individuals are excluded.*

For the elephant seal data, there were several differences in parameter estimates of the JSTL model when recycled individuals were included compared to when recycled individuals were excluded. For this analysis, we used the same model as the simulation study where capture, survival, and tag‐retention rates were held constant.

As expected, the super‐population size estimate for the analysis which included the recycled individuals $\left(\hat{N}=8,985\right)$ is 30% larger than the estimate in the analysis which excluded recycled individuals $\left(\hat{N}=6,949\right)$ who were recognized upon recapture. This relationship also holds true for the abundance estimates at each sample time (Table 2). The difference in the abundance estimates increases as time goes on, again validating the results of our simulation study. Standard error estimates for ${\hat{N}}_{t}$ are also higher when recycled individuals are included in the analysis. The same pattern is seen in the simulation studies (see Appendix S1).

**Table 2:** Estimates of survival probability (*ɸ*), capture probability (*p*), tag‐retention probability (*λ*), and annual population size (*N_j_*) for the elephant seal data analyzed including and excluding recycled individuals

Parameter	Including recycled		Excluding recycled	
	Estimate	*SE*	Estimate	*SE*
*ϕ*	0.759	0.006	0.744	0.006
*p*	0.682	0.006	0.741	0.006
*λ*	0.792	0.005	0.799	0.005
*N*_1994_	1,740	48	1,601	36
*N*_1995_	1,859	41	1,717	40
*N*_1996_	2,515	46	2,264	42
*N*_1997_	3,179	50	2727	43
*N*_1998_	3,793	54	2,965	48
*N*_1999_	4,300	59	3,229	46
*N*_2000_	4,973	65	3,238	50
N	8,985		6,949	

Note

Estimated standard errors (*SE*) are also presented.

Similar to the simulations, there is not much difference in the estimates of survival, capture, and tag‐retention probabilities between the analysis including and excluding recycled individuals. For comparison to the previous simulations, the tag‐retention probability for the elephant seals is estimated to be ≈0.8 (high).

## 5 DISCUSSION

Through both a simulation study and an elephant seal case study, we examined the effect of recycled individuals on parameter estimates from the Jolly–Seber tag loss model. In an attempt to emulate the many different real‐life scenarios researchers may face, we simulated over many different values of survival probability, capture probability, tag‐retention probability, population size, study length, and proportion double‐tagged. While these scenarios do not cover all possible realistic mark‐recapture experiments, our simulations are sufficient to show that the JSTL abundance estimates can be substantially biased by recycled individuals, especially when tag‐retention is low combined with high survival, high capture rates, or both. This effect is especially noticeable in longer experiments. These results bring context to the assumption that the effect of recycled individuals is negligible in mark‐recapture models. However, we show that in general, recycled individuals have little effect on the accuracy of the survival, capture, and tag‐retention probability estimates and that for short‐term studies, the effects are reduced.

For longer term studies when survival and capture probabilities are low, the bias in abundance estimates associated with recycled individuals is smaller. These are characteristics that might be associated with small, endangered, or decreasing populations.

As expected, the survival estimates are typically unbiased when recycled individuals are excluded. Survival estimates are essentially a relative measure of how many individuals are around now versus the previous time step. Thus, the bias in the numerator and the denominator essentially cancels out (i.e., ${\hat{\phi }}_{j}\approx \hat{N}/{\hat{N}}_{j-1}$). Although the case study of elephant seals validated some of the results from the simulation study (recycled individuals bias abundance estimates upwards), some caution must be taken when comparing simulation studies to the real world. There are many parameters that may differ or be uncertain, such as entry probabilities, that may influence the results. Simplifications of the individuals in the simulation studies may not take into account the complexities that arise in real‐life scenarios.

Although our study provides some evidence that recycled individuals have an effect on estimators of the JSTL model in particular situations, there is room for improvement in our approach and questions remain for future work. We only examined three levels of survival, capture, and tag‐retention probabilities (low = 0.2, medium = 0.5, and high = 0.9) which was intended to simulate across a variety of scenarios that may exist in real life. For researchers with a particular population in mind, different levels of survival, capture, or tag retention could be investigated. Additionally, future work could examine the effect of recycled individuals in situations where survival, capture, or tag‐retention probabilities are thought to be time‐ or group‐varying.

Developing a model to incorporate recycled individuals is a similar problem to that of incorporating misidentification of individuals. Schwarz and Stobo (1999) developed a model to deal with tag misreads in an open population capture–recapture setting. However, most of the misidentification literature focusses on genetic or photographic identification errors. Here, multiple identities can be assigned to the same individual leading to overestimates in population size if misidentification is ignored (Yoshizaki, Brownie, Pollock, & Link, 2011). This is the same result that we see when recycled individuals are ignored. Link, Yoshizaki, Bailey, and Pollock (2010) introduced the notion of using a latent multinomial to model the latent capture histories for a closed population model. Others have extended Link et al.'s model to deal with multiple noninvasive marks (Bonner & Holmberg, 2013; McClintock, Conn, Alonso, & Crooks, 2013), heterogeneity in parameters (McClintock, Bailey, Dreher, & Link, 2014) and open populations (Bonner & Holmberg, 2013). These latent multinomial models could be extended to include misidentification produced by complete tag loss.

Finally, the JSTL model we used did not include a component for loss on capture (when for example a fishery harvest occurs). It would be interesting for future work to include loss on capture to determine whether recycled individuals are still problematic under this scenario. Increasing computation power and a larger community applying themselves to these problems has the potential to inform researchers and managers in a meaningful way, especially in terms of how we use imperfect observations to estimate vital rates (survival and fecundity). Having more robust estimates of vital rates is especially important if we are to effectively manage populations on an ever increasing list of endangered species.

For researchers interested in conducting and analyzing mark‐recapture studies to determine abundance estimates, we stress the importance of using tags with high retention rates, especially in situations where survival and capture rates are suspected to be high. As long as tag retention is high, the JSTL estimator of population size is only weakly affected by recycled individuals. Longer studies should be particularly concerned about recycled individuals biasing abundance estimates. In situations where it is possible, recognizing whether an individual has been captured previously (by scarring, marking, etc) can improve accuracy of the abundance estimates. Permanent marking should be used where possible. If researchers are only interested in the survival rates, they do not need to be concerned with the effect of recycled individuals regardless of the study's tag‐retention rates.

Alternatively, researchers could replace lost tags on a recaptured individual thereby minimizing the occurrence of complete tag loss. Depending on model assumptions, the JSTL model may not be appropriate for a study design involving retagging. Future work would involve extending the JSTL model to incorporate retagged individuals and assess the performance of recycled individuals within this framework.

## CONFLICT OF INTEREST

None declared.

## AUTHORS' CONTRIBUTIONS

EMW and LC conceived the ideas, designed methodology, and analyzed the data; CM collected the data. All authors led the writing of the manuscript. All authors contributed critically to the drafts and gave final approval for publication.

## Data Availability

Data and R scripts available from the Dryad Digital Repository: https://doi.org/10.5061/dryad.v6wwpzgr3
