# Supplementary material for: Complete tag loss in capture–recapture studies affects abundance estimates: An elephant seal case study
Source: Ecol Evol. 2020 Jan 30;10(5):2377–84. doi: 10.1002/ece3.6052 (PMC7069293; doi:10.1002/ece3.6052)
Supplement: Supplementary file 1 [file ECE3-10-2377-s001.pdf]

Web-based Supplementary Materials for Complete tag loss in capture-recapture studies affects abundance estimates: an elephant seal case study

Web Appendix A: The Jolly-Seber Tag Loss Model

We provide the assumptions and and detailed notation for the Jolly-Seber tag loss (JSTL) model under constant survival, capture, and tag retention probabilities, and time-varying entry probabilities. Although Cowen and Schwarz (2006) provide for loss on capture in the original JSTL model (due to a fishery or harvest for example), we have removed this component from the likelihood and it is not discussed in this development.

Assumptions

Assumptions of the JSTL model with constant survival, capture, and tag retention probabilities and time-varying entry probabilities are as follows:

- The effect of recycled individuals is negligible.
- All individuals (marked and unmarked) are equally catchable and capture probabilities for all individuals are the same for all individuals at all sample time.
- All individuals (marked and unmarked) have equal survival probabilities between all sample times.
- All individuals have equal entry (birth or immigration) probabilities, but entry probabilities can vary between sample times.
- All marked individuals have equal tag retention probabilities between all sample times.
- For double-tagged individuals, tag loss is independent between tags.
- There is independence across all individuals.
- The sampling period is relatively short compared to the interval between sampling times.
- Recycled individuals are negligible.

Notation

We use the following notation to describe the statistics or model parameters discussed in this study.

Statistics:

$k =$ number of sample times
$m =$ number of unique tag histories
$n_{\text{obs}} =$ the total number of unique individuals observed throughout the study
$nt_j =$ number of tags on the individuals at sample time j, $j = 1, 2, \cdots, k$
$f_i =$ first sample time individual i was captured
$l_{id} =$ last sample time individual i was captured with tag d present, $d = 1, 2$
$l_i =$ last sample time where individual i was captured
$\omega^*_{ij} =$ component of the capture history vector $i = 1, 2, \cdots, m$

$$\omega^*_{ij} = \begin{cases} 1 & \text{if individual } i \text{ was captured for the first time at sample time } f_i \\ 1 & \text{if individual } i \text{ was captured at sample time } f_i < j \text{ with at least one tag present} \\ 0 & \text{if individual } i \text{ was not captured at sample time } j \end{cases}$$

$q_{id} =$ first sample time where tag d was known to be missing for individual i

Parameters:

$p =$ the probability that an individual is recaptured given that the individual was alive at the previous sample time

$\phi =$ the probability that an individual survives and remains in the population between a sample times and the next sample time, given it was alive and in the population at the previous sample time

$b_j =$ the probability that an individual enters the system between sample times j and $j+1$. $j = 0, 1, ..., k-1$. b_0 is the expected fraction of individuals alive just prior to the first sample time, with $\sum_{j=0}^{k-1} b_j = 1$

$T_d =$ the probability that an individual is marked with d tags. Note that the probability of marking with a single tag is one minus the probability of marking with a double tag: $T_1 = 1 - T_2$

$\lambda =$ the probability that an individual captured will retain its tag between sample times given that it remains alive

$N =$ super-population size, which is the total number of individuals ever present in population and available for capture during the study

Functions of Parameters:

$\chi_{(f_i, l_i, nt)} =$ the probability that the individual with capture history i is first seen at f_i and not seen after sample time l_i, with nt tags. This is a recursive function of ϕ, p, and λ. If $f_i = 0$, this indicates individuals not yet captured but alive at time l_i.

For individuals not yet captured:

$$\chi_{(0,j,0)} = \begin{cases} 1 - \phi + \phi(1-p)\chi_{(0,j+1,0)} & \text{if } j < k \\ 1 & \text{if } j = k \end{cases}$$

For single tagged individuals:

$$\chi_{(f_i,j,1)} = \begin{cases} 1 - \phi + \phi(1-p)\lambda\chi_{(f_i,j+1,1)} + \phi(1-\lambda) & \text{if } j < k \\ 1 & \text{if } j = k \end{cases}$$

For double tagged individuals:

$$\chi_{(f_i,j,2)} = \begin{cases} 1 - \phi + \phi(1-p)\lambda^2\chi_{(f_i,j+1,2)} + \phi(1-\lambda)^2 + 2\phi(1-p)\lambda(1-\lambda)\chi_{(f_i,j+1,1)} & \text{if } j < k \\ 1 & \text{if } j = k \end{cases}$$

$\psi_j =$ probability that an individual enters the population, is still alive, and is not seen before time j;

$$\psi_j = \begin{cases} b_0 & \text{if } j = 1 \\ \psi_j(1-p_j)\phi_j + b_j & \text{if } j = 2, ..., k \end{cases}$$

$B_j =$ net births; the number of individuals who enter the population after sample time j and survive to sample time $j+1$; $j = 0, 1, ..., k-1$. B_0 is the number of individuals alive just before the first sample time. Note that $E(B_j|N) = Nb_j$.

$N_j =$ population size at time j. $\text{E}(N_1|N) = B_0$, $\text{E}(N_{j+1}|N) = N_j\phi + B_j$, which is the number of individuals that survive from time j plus the number of births.

Likelihood

The full likelihood can be written as:

$$L = \binom{N}{n_{\text{obs}}} \left\{ \sum_{j=0}^{k-1} b_j(1-p)\chi_{(0,j+1,0)} \right\}^{(N-n_{\text{obs}})} \times \left\{ 1 - \sum_{j=0}^{k-1} b_j(1-p)\chi_{(0,j+1,0)} \right\}^{n_{\text{obs}}} \times$$

$$\binom{n_{\text{obs}}}{n_{\omega_1}, n_{\omega_2}, ..., n_{\omega_m}} \prod_{i=1}^{m} \left[\psi_{f_i} T_d \left\{ \prod_{j=f_i}^{l_i} p^{w*_{ij}} (1-p)^{(1-w*_{ij})} \right\} \left\{ \prod_{j=f_i}^{l_i-1} \phi \right\} \times \right.$$

$$\left. \prod_{d=1}^{2} \left\{ \left(\prod_{j=f_i}^{l_{id}-1} \lambda \right) \left(1 - \prod_{j=l_{id}}^{q_{id}-1} \lambda \right)^{I(l_{id} \neq l_i)} \right\} \times \chi_{f_i, l_i, nt_{l_i}} \right]^{n_{\omega_i}} \left\{ 1 - \sum_{j=0}^{k-1} b_j(1-p)\chi_{(0,j+1,0)} \right\}^{-n_{\text{obs}}}$$

Survival Estimates

N=1000, T=1

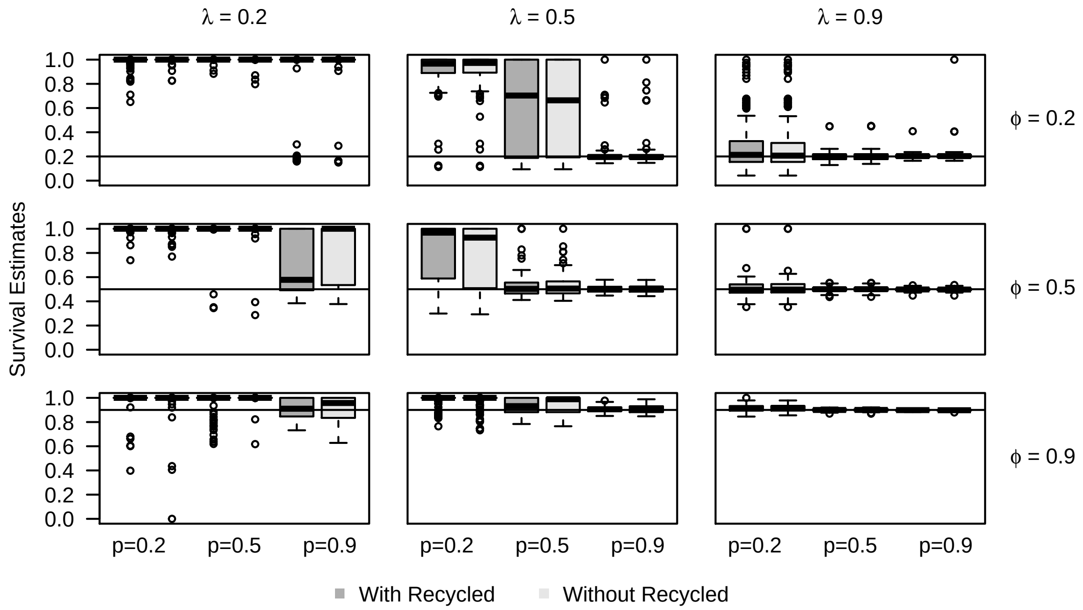

WEB FIGURE 1: Boxplots of survival estimates ($\hat{\phi}$) of 100 simulated datasets analyzed including and excluding the effect of recycled individuals for population size 1000 with $T_2 = 1$ with 10 sample times for varying survival ($\phi = 0.2, 0.5, 0.9$), capture ($p = 0.2, 0.5, 0.9$), and tag retention ($\lambda = 0.2, 0.5, 0.9$) probabilities. The black line indicates the true value of ϕ used to simulate the data for each model.

WEB TABLE 1: Standard Error (SE), Standard Deviation (SD), and Root Mean Squared Error (RMSE) of survival estimates ($\hat{\phi}$) of 100 simulated datasets analyzed excluding (R') and including(R) the effect of recycled individuals with population size $N = 1000$ with $T_2 = 1$ with 10 sample times for varying tag retention ($\lambda = 0.2, 0.5, 0.9$), varying survival probabilities ($\phi = 0.2, 0.5, 0.9$) and varying capture probabilities ($p = 0.2, 0.5, 0.9$).

ϕ	p	λ	SE (R')	SE (R)	SD (R')	SD (R)	RMSE (R')	RMSE (R)
0.9	0.9	0.9	0.0066	0.0064	0.0066	0.0064	0.0069	0.0066
0.9	0.9	0.5	0.0334	0.0241	0.0327	0.0243	0.0337	0.0249
0.9	0.9	0.2	0.0796	0.0620	0.0926	0.0792	0.0941	0.0797
0.9	0.5	0.9	0.0095	0.0093	0.0097	0.0093	0.0096	0.0093
0.9	0.5	0.5	0.0286	0.0332	0.0683	0.0622	0.0801	0.0691
0.9	0.5	0.2	0.0039	0.0336	0.0420	0.0939	0.1032	0.1107
0.9	0.2	0.9	0.0224	0.0224	0.0249	0.0274	0.0291	0.0305
0.9	0.2	0.5	0.0213	0.0180	0.0620	0.0483	0.0963	0.0944
0.9	0.2	0.2	0.0117	0.0150	0.0837	0.0929	0.1190	0.1214
0.5	0.9	0.9	0.0138	0.0137	0.0132	0.0129	0.0132	0.0129
0.5	0.9	0.5	0.0307	0.0277	0.0317	0.0299	0.0318	0.0298
0.5	0.9	0.2	0.0544	0.0699	0.2501	0.2416	0.3815	0.3098
0.5	0.5	0.9	0.0212	0.0211	0.0217	0.0218	0.0216	0.0217
0.5	0.5	0.5	0.0554	0.0511	0.0969	0.1099	0.1002	0.1130
0.5	0.5	0.2	0.0059	0.0100	0.0934	0.1059	0.4943	0.4928
0.5	0.2	0.9	0.0465	0.0465	0.0870	0.0871	0.0880	0.0875
0.5	0.2	0.5	0.0651	0.0630	0.2466	0.2362	0.3861	0.3940
0.5	0.2	0.2	0.0135	0.0076	0.0334	0.0303	0.4929	0.4955
0.2	0.9	0.9	0.0147	0.0147	0.0857	0.0257	0.0865	0.0260
0.2	0.9	0.5	0.0264	0.0254	0.1393	0.1262	0.1420	0.1280
0.2	0.9	0.2	0.0058	0.0071	0.1597	0.2061	0.7824	0.7709
0.2	0.5	0.9	0.0310	0.0308	0.0507	0.0448	0.0508	0.0446
0.2	0.5	0.5	0.0481	0.0500	0.3683	0.3729	0.5306	0.5476
0.2	0.5	0.2	0.0039	0.0042	0.0289	0.0154	0.7955	0.7976
0.2	0.2	0.9	0.0692	0.0701	0.2481	0.2485	0.2661	0.2682
0.2	0.2	0.5	0.0586	0.0677	0.1722	0.1681	0.7256	0.7278
0.2	0.2	0.2	0.0086	0.0213	0.0266	0.0563	0.7951	0.7848

N=100000, T=1

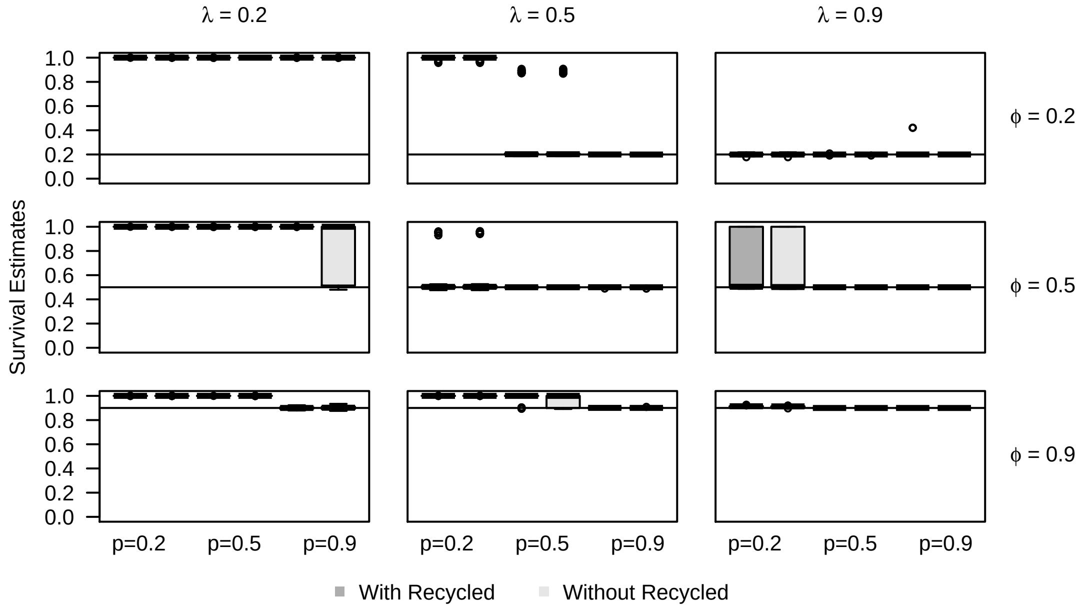

Web Figure 2: Boxplots of survival estimates ($\hat{\phi}$) of 100 simulated datasets analyzed including and excluding the effect of recycled individuals for population size 100000 with $T_2 = 1$ with 10 sample times for varying survival ($\phi = 0.2, 0.5, 0.9$), capture ($p = 0.2, 0.5, 0.9$), and tag retention ($\lambda = 0.2, 0.5, 0.9$) probabilities. The black line indicates the true value of ϕ used to simulate the data for each model.

Web Table 2: Standard Error (SE), Standard Deviation (SD), and Root Mean Squared Error (RMSE) of survival estimates ($\hat{\phi}$) of 100 simulated datasets analyzed excluding (R') and including(R) the effect of recycled individuals with population size $N = 100000$ with $T_2 = 1$ with 10 sample times for varying tag retention ($\lambda = 0.2, 0.5, 0.9$), varying survival probabilities ($\phi = 0.2, 0.5, 0.9$) and varying capture probabilities ($p = 0.2, 0.5, 0.9$).

ϕ	p	λ	SE (R')	SE (R)	SD (R')	SD (R)	RMSE (R')	RMSE (R)
0.9	0.9	0.9	0.0007	0.0006	0.0006	0.0006	0.0006	0.0006
0.9	0.9	0.5	0.0032	0.0024	0.0031	0.0023	0.0031	0.0024
0.9	0.9	0.2	0.0138	0.0083	0.0136	0.0088	0.0135	0.0087
0.9	0.5	0.9	0.0009	0.0009	0.0009	0.0009	0.0009	0.0009
0.9	0.5	0.5	0.0048	0.0039	0.0510	0.0342	0.0742	0.0933
0.9	0.5	0.2	0.0000	0.0000	0.0000	0.0000	0.1000	0.1000
0.9	0.2	0.9	0.0022	0.0022	0.0027	0.0025	0.0132	0.0144
0.9	0.2	0.5	0.0001	0.0000	0.0000	0.0000	0.1000	0.1000
0.9	0.2	0.2	0.0000	0.0000	0.0000	0.0000	0.1000	0.1000
0.5	0.9	0.9	0.0014	0.0014	0.0014	0.0014	0.0014	0.0014
0.5	0.9	0.5	0.0030	0.0028	0.0032	0.0028	0.0032	0.0028
0.5	0.9	0.2	0.0100	0.0000	0.2228	0.0000	0.4272	0.5000
0.5	0.5	0.9	0.0021	0.0021	0.0021	0.0021	0.0021	0.0021
0.5	0.5	0.5	0.0054	0.0051	0.0055	0.0056	0.0054	0.0056
0.5	0.5	0.2	0.0000	0.0000	0.0000	0.0000	0.5000	0.5000
0.5	0.2	0.9	0.0033	0.0028	0.2351	0.2479	0.2829	0.3202
0.5	0.2	0.5	0.0123	0.0120	0.1297	0.1157	0.1357	0.1197
0.5	0.2	0.2	0.0001	0.0001	0.0000	0.0000	0.5000	0.5000
0.2	0.9	0.9	0.0015	0.0015	0.0014	0.0311	0.0014	0.0312
0.2	0.9	0.5	0.0025	0.0024	0.0026	0.0025	0.0026	0.0026
0.2	0.9	0.2	0.0000	0.0000	0.0000	0.0000	0.8000	0.8000
0.2	0.5	0.9	0.0031	0.0031	0.0032	0.0032	0.0032	0.0032
0.2	0.5	0.5	0.0064	0.0065	0.2382	0.2703	0.2556	0.2988
0.2	0.5	0.2	0.0002	0.0001	0.0000	0.0000	0.8000	0.8000
0.2	0.2	0.9	0.0074	0.0074	0.0074	0.0074	0.0073	0.0073
0.2	0.2	0.5	0.0070	0.0065	0.0117	0.0109	0.7946	0.7953
0.2	0.2	0.2	0.0002	0.0002	0.0000	0.0000	0.8000	0.8000

N=1000, T=0.5

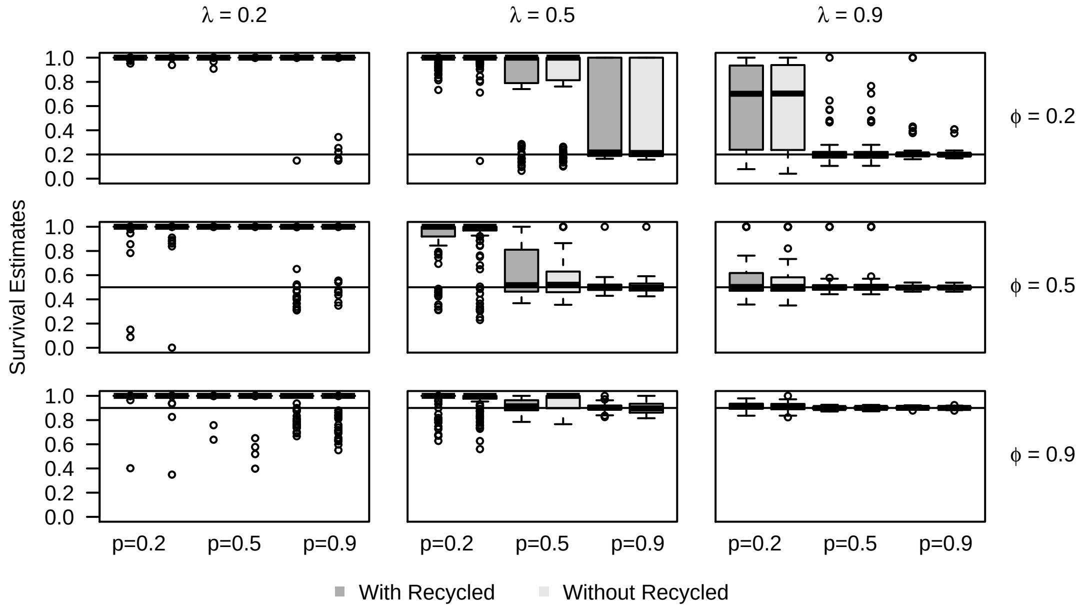

Web Figure 3: Boxplots of survival estimates ($\hat{\phi}$) of 100 simulated datasets analyzed including and excluding the effect of recycled individuals for population size 1000 with $T_2 = 0.5$ with 10 sample times for varying survival ($\phi = 0.2, 0.5, 0.9$), capture ($p = 0.2, 0.5, 0.9$), and tag retention ($\lambda = 0.2, 0.5, 0.9$) probabilities. The black line indicates the true value of ϕ used to simulate the data for each model.

Web Table 3: Standard Error (SE), Standard Deviation (SD), and Root Mean Squared Error (RMSE) of survival estimates ($\hat{\phi}$) of 100 simulated datasets analyzed excluding (R') and including(R) the effect of recycled individuals with population size $N = 1000$ with $T_2 = 0.5$ with 10 sample times for varying tag retention ($\lambda = 0.2, 0.5, 0.9$), varying survival probabilities ($\phi = 0.2, 0.5, 0.9$) and varying capture probabilities ($p = 0.2, 0.5, 0.9$).

ϕ	p	λ	SE (R')	SE (R)	SD (R')	SD (R)	RMSE (R')	RMSE (R)
0.9	0.9	0.9	0.0082	0.0077	0.0085	0.0080	0.0085	0.0081
0.9	0.9	0.5	0.0459	0.0319	0.0458	0.0311	0.0456	0.0312
0.9	0.9	0.2	0.0294	0.0389	0.1177	0.0939	0.1262	0.1069
0.9	0.5	0.9	0.0115	0.0111	0.0109	0.0110	0.0109	0.0110
0.9	0.5	0.5	0.0257	0.0462	0.0725	0.0589	0.0888	0.0611
0.9	0.5	0.2	0.0063	0.0044	0.0932	0.0434	0.1234	0.1034
0.9	0.2	0.9	0.0256	0.0256	0.0312	0.0307	0.0332	0.0335
0.9	0.2	0.5	0.0311	0.0233	0.0916	0.0935	0.1063	0.1106
0.9	0.2	0.2	0.0066	0.0041	0.0676	0.0599	0.1128	0.1110
0.5	0.9	0.9	0.0151	0.0148	0.0164	0.0160	0.0164	0.0160
0.5	0.9	0.5	0.0410	0.0361	0.0646	0.0615	0.0645	0.0614
0.5	0.9	0.2	0.0109	0.0203	0.1496	0.2041	0.4801	0.4628
0.5	0.5	0.9	0.0215	0.0215	0.1396	0.1227	0.1437	0.1249
0.5	0.5	0.5	0.0693	0.0534	0.1787	0.2249	0.1971	0.2548
0.5	0.5	0.2	0.0004	0.0003	0.0000	0.0000	0.5000	0.5000
0.5	0.2	0.9	0.0468	0.0459	0.1756	0.1849	0.1891	0.2002
0.5	0.2	0.5	0.0390	0.0376	0.2110	0.1979	0.4543	0.4481
0.5	0.2	0.2	0.0094	0.0104	0.0279	0.1262	0.4945	0.4942
0.2	0.9	0.9	0.0158	0.0154	0.0372	0.1430	0.0373	0.1453
0.2	0.9	0.5	0.0303	0.0251	0.3840	0.3924	0.4876	0.5078
0.2	0.9	0.2	0.0073	0.0008	0.1702	0.0851	0.7799	0.7960
0.2	0.5	0.9	0.0326	0.0325	0.0976	0.1181	0.0982	0.1197
0.2	0.5	0.5	0.0405	0.0348	0.3305	0.3247	0.6892	0.6895
0.2	0.5	0.2	0.0008	0.0027	0.0000	0.0097	0.8000	0.7988
0.2	0.2	0.9	0.0644	0.0661	0.3416	0.3369	0.5360	0.5223
0.2	0.2	0.5	0.0273	0.0278	0.0952	0.0455	0.7811	0.7839
0.2	0.2	0.2	0.0009	0.0028	0.0061	0.0057	0.7994	0.7991

N=100000, T=0.5

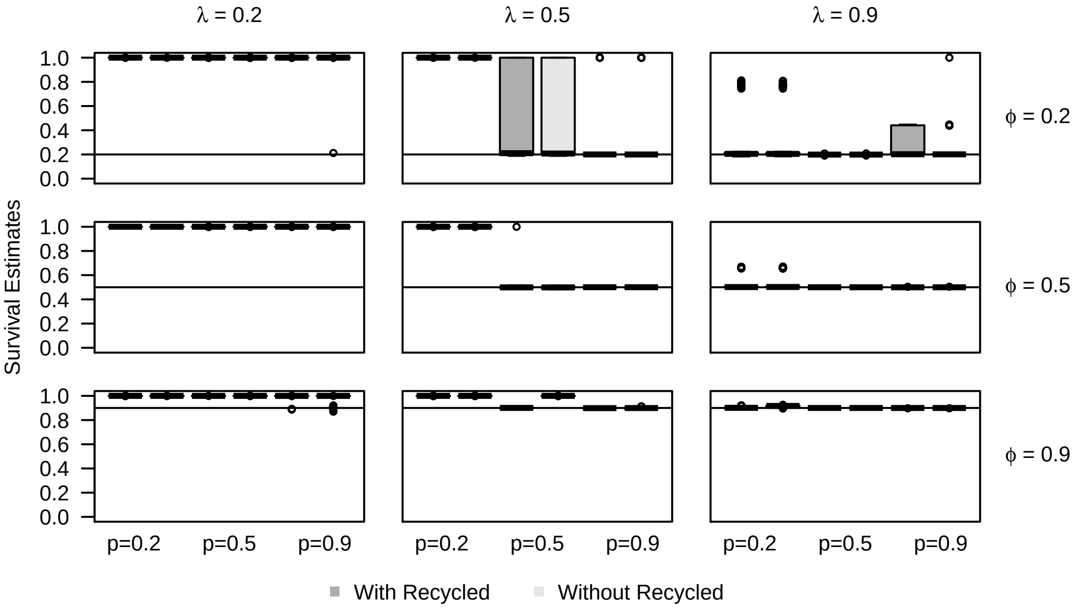

Web Figure 4: Boxplots of survival estimates ($\hat{\phi}$) of 100 simulated datasets analyzed including and excluding the effect of recycled individuals for population size 100000 with $T_2 = 0.5$ with 10 sample times for varying survival ($\phi = 0.2, 0.5, 0.9$), capture ($p = 0.2, 0.5, 0.9$), and tag retention ($\lambda = 0.2, 0.5, 0.9$) probabilities. The black line indicates the true value of ϕ used to simulate the data for each model.

Web Table 4: Standard Error (SE), Standard Deviation (SD), and Root Mean Squared Error (RMSE) of survival estimates ($\hat{\phi}$) of 100 simulated datasets analyzed excluding (R') and including(R) the effect of recycled individuals with population size $N = 100000$ with $T_2 = 0.5$ with 10 sample times for varying tag retention ($\lambda = 0.2, 0.5, 0.9$), varying survival probabilities ($\phi = 0.2, 0.5, 0.9$) and varying capture probabilities ($p = 0.2, 0.5, 0.9$).

ϕ	p	λ	SE (R')	SE (R)	SD (R')	SD (R)	RMSE (R')	RMSE (R)
0.9	0.9	0.9	0.0008	0.0008	0.0009	0.0009	0.0009	0.0009
0.9	0.9	0.5	0.0045	0.0032	0.0052	0.0033	0.0052	0.0034
0.9	0.9	0.2	0.0179	0.0073	0.0343	0.0155	0.0932	0.0988
0.9	0.5	0.9	0.0012	0.0011	0.0010	0.0010	0.0010	0.0010
0.9	0.5	0.5	0.0000	0.0055	0.0000	0.0052	0.1000	0.0052
0.9	0.5	0.2	0.0000	0.0000	0.0000	0.0001	0.1000	0.0999
0.9	0.2	0.9	0.0026	0.0026	0.0055	0.0060	0.0165	0.0064
0.9	0.2	0.5	0.0000	0.0000	0.0000	0.0000	0.1000	0.1000
0.9	0.2	0.2	0.0000	0.0000	0.0000	0.0000	0.1000	0.1000
0.5	0.9	0.9	0.0015	0.0015	0.0014	0.0014	0.0014	0.0014
0.5	0.9	0.5	0.0041	0.0036	0.0041	0.0037	0.0041	0.0037
0.5	0.9	0.2	0.0000	0.0000	0.0000	0.0000	0.5000	0.5000
0.5	0.5	0.9	0.0023	0.0023	0.0020	0.0020	0.0020	0.0020
0.5	0.5	0.5	0.0068	0.0064	0.0068	0.0510	0.0068	0.0509
0.5	0.5	0.2	0.0000	0.0000	0.0000	0.0000	0.5000	0.5000
0.5	0.2	0.9	0.0052	0.0051	0.0566	0.0442	0.0612	0.0463
0.5	0.2	0.5	0.0000	0.0000	0.0000	0.0000	0.5000	0.5000
0.5	0.2	0.2	0.0002	0.0002	0.0000	0.0000	0.5000	0.5000
0.2	0.9	0.9	0.0018	0.0020	0.1240	0.1154	0.1367	0.1414
0.2	0.9	0.5	0.0031	0.0029	0.2704	0.2871	0.2885	0.3098
0.2	0.9	0.2	0.0005	0.0000	0.0788	0.0000	0.7960	0.8000
0.2	0.5	0.9	0.0033	0.0033	0.0032	0.0032	0.0033	0.0032
0.2	0.5	0.5	0.0064	0.0063	0.3971	0.4020	0.5184	0.5484
0.2	0.5	0.2	0.0001	0.0001	0.0000	0.0000	0.8000	0.8000
0.2	0.2	0.9	0.0085	0.0086	0.2210	0.2344	0.2438	0.2634
0.2	0.2	0.5	0.0007	0.0007	0.0001	0.0001	0.7999	0.7999
0.2	0.2	0.2	0.0003	0.0003	0.0000	0.0000	0.8000	0.8000

N=1000, T=1, t=5

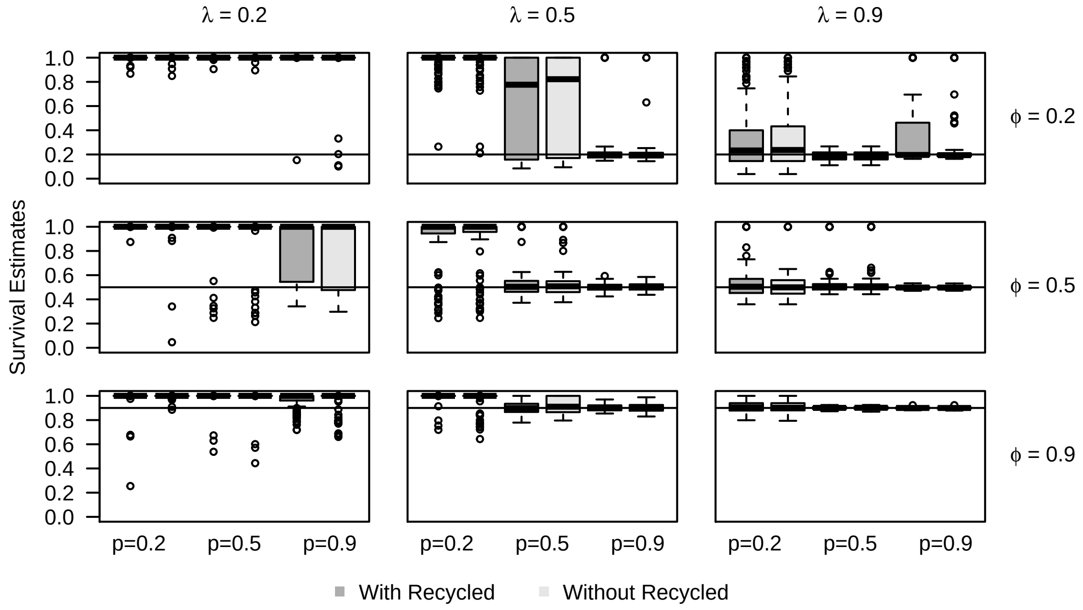

Web Figure 5: Boxplots of survival estimates ($\hat{\phi}$) of 100 simulated datasets analyzed including and excluding the effect of recycled individuals for population size 1000 with $T_2 = 1$ with 5 sample times for varying survival ($\phi = 0.2, 0.5, 0.9$), capture ($p = 0.2, 0.5, 0.9$), and tag retention ($\lambda = 0.2, 0.5, 0.9$) probabilities. The black line indicates the true value of ϕ used to simulate the data for each model.

N=1000, T=1, t=7

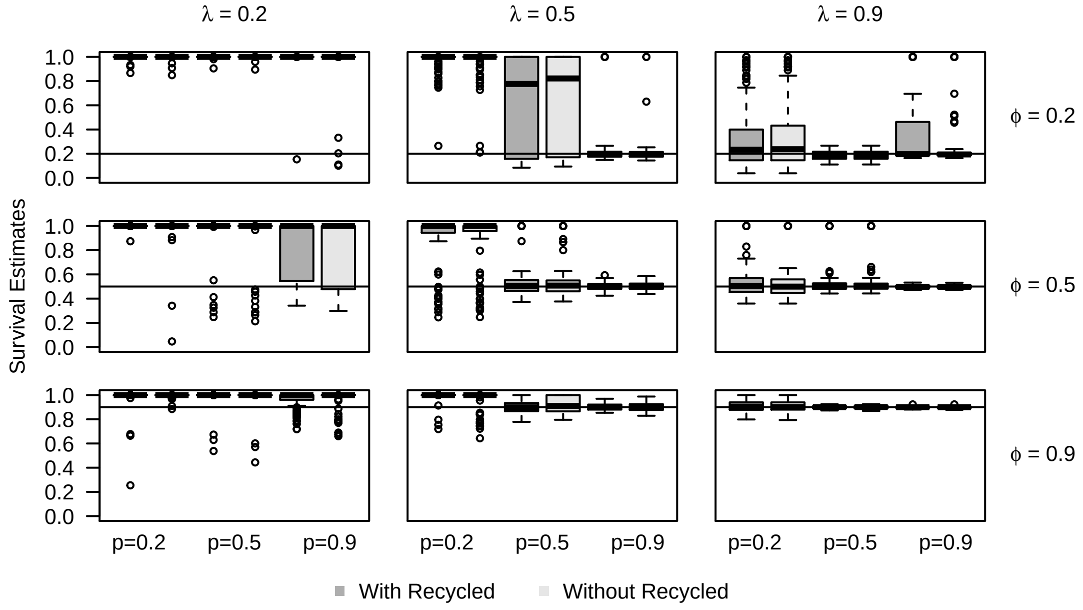

Web Figure 6: Boxplots of survival estimates ($\hat{\phi}$) of 100 simulated datasets analyzed including and excluding the effect of recycled individuals for population size 1000 with $T_2 = 1$ with 7 sample times for varying survival ($\phi = 0.2, 0.5, 0.9$), capture ($p = 0.2, 0.5, 0.9$), and tag retention ($\lambda = 0.2, 0.5, 0.9$) probabilities. The black line indicates the true value of ϕ used to simulate the data for each model.

Capture Estimates

N=1000, T=1

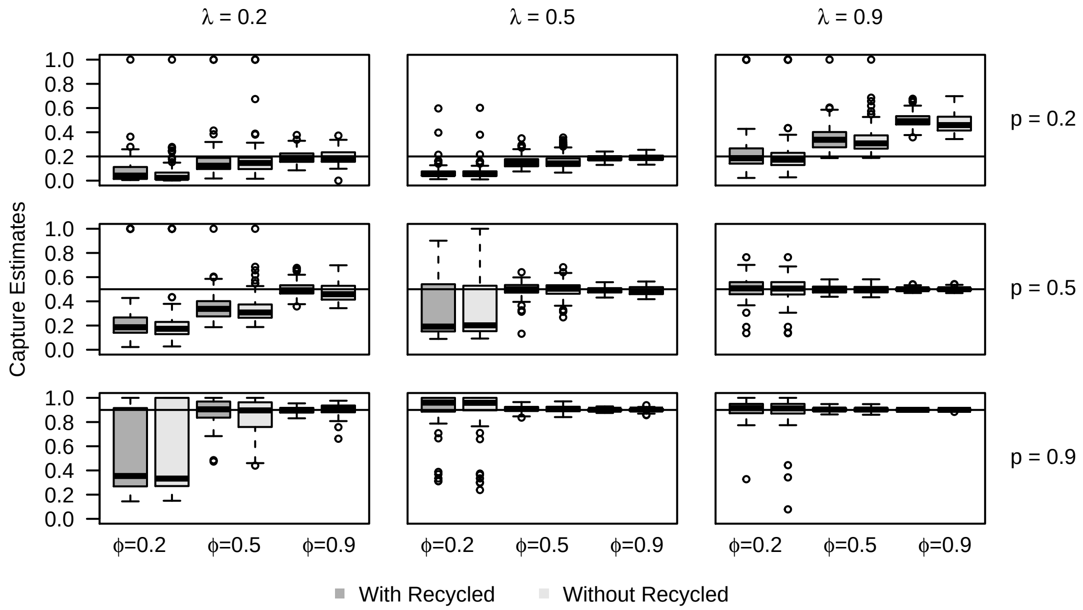

Web Figure 7: Boxplots of capture estimates ($\hat{p}$) of 100 simulated datasets analyzed including and excluding the effect of recycled individuals for population size 1000 with $T_2 = 1$ with 10 sample times for varying survival ($\phi = 0.2, 0.5, 0.9$), capture ($p = 0.2, 0.5, 0.9$), and tag retention ($\lambda = 0.2, 0.5, 0.9$) probabilities. The black line indicates the true value of p used to simulate the data for each model.

Web Table 5: Standard Error (SE), Standard Deviation (SD), and Root Mean Squared Error (RMSE) of capture estimates ($\hat{p}$)of 100 simulated datasets analyzed excluding (R') and including(R) the effect of recycled individuals with population size $N = 1000$ with $T_2 = 1$ with 10 sample times for varying tag retention ($\lambda = 0.2, 0.5, 0.9$), varying survival probabilities ($\phi = 0.2, 0.5, 0.9$) and varying capture probabilities ($p = 0.2, 0.5, 0.9$).

ϕ	p	λ	SE (R')	SE (R)	SD (R')	SD (R)	RMSE (R')	RMSE (R)
0.9	0.9	0.9	0.0066	0.0065	0.0063	0.0060	0.0063	0.0060
0.9	0.9	0.5	0.0139	0.0106	0.0147	0.0116	0.0146	0.0116
0.9	0.9	0.2	0.0374	0.0245	0.0484	0.0251	0.0484	0.0251
0.9	0.5	0.9	0.0131	0.0129	0.0151	0.0148	0.0150	0.0147
0.9	0.5	0.5	0.0251	0.0213	0.0343	0.0269	0.0365	0.0283
0.9	0.5	0.2	0.0574	0.0470	0.0746	0.0590	0.0793	0.0587
0.9	0.2	0.9	0.0147	0.0147	0.0167	0.0178	0.0180	0.0186
0.9	0.2	0.5	0.0227	0.0203	0.0250	0.0225	0.0267	0.0260
0.9	0.2	0.2	0.0530	0.0470	0.0557	0.0514	0.0555	0.0515
0.5	0.9	0.9	0.0167	0.0166	0.0172	0.0172	0.0174	0.0173
0.5	0.9	0.5	0.0275	0.0251	0.0262	0.0256	0.0270	0.0264
0.5	0.9	0.2	0.0592	0.0570	0.1815	0.1119	0.1974	0.1128
0.5	0.5	0.9	0.0323	0.0322	0.0331	0.0329	0.0330	0.0328
0.5	0.5	0.5	0.0526	0.0498	0.0665	0.0680	0.0662	0.0678
0.5	0.5	0.2	0.0717	0.0696	0.1207	0.1175	0.1979	0.1852
0.5	0.2	0.9	0.0339	0.0342	0.0462	0.0457	0.0459	0.0455
0.5	0.2	0.5	0.0385	0.0357	0.0631	0.0562	0.0730	0.0709
0.5	0.2	0.2	0.0621	0.0536	0.1698	0.1628	0.1704	0.1650
0.2	0.9	0.9	0.0426	0.0428	0.1209	0.0769	0.1205	0.0768
0.2	0.9	0.5	0.0467	0.0489	0.1644	0.1473	0.1636	0.1466
0.2	0.9	0.2	0.0673	0.0785	0.3190	0.3079	0.5023	0.4954
0.2	0.5	0.9	0.0916	0.0926	0.0997	0.0952	0.0992	0.0951
0.2	0.5	0.5	0.0795	0.0789	0.2462	0.2420	0.2888	0.2873
0.2	0.5	0.2	0.0626	0.0655	0.2080	0.2367	0.3364	0.3405
0.2	0.2	0.9	0.0912	0.0895	0.2011	0.2017	0.2021	0.2023
0.2	0.2	0.5	0.0310	0.0318	0.0714	0.0716	0.1487	0.1480
0.2	0.2	0.2	0.0266	0.0384	0.1145	0.1184	0.1792	0.1698

N=100000, T=1

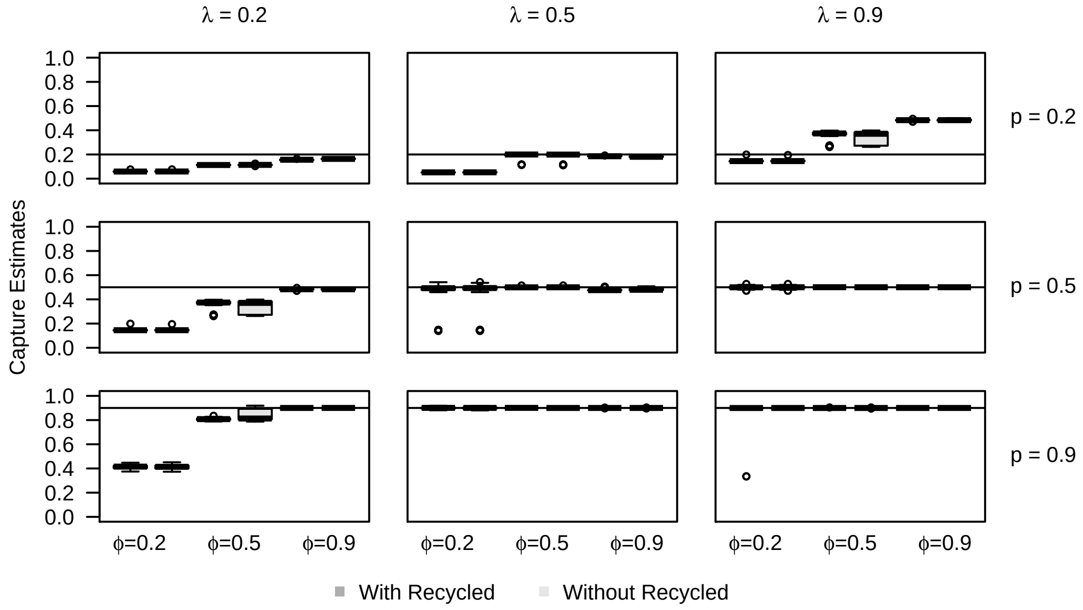

Web Figure 8: Boxplots of capture estimates ($\hat{p}$) of 100 simulated datasets analyzed including and excluding the effect of recycled individuals for population size 100000 with $T_2 = 1$ with 10 sample times for varying survival ($\phi = 0.2, 0.5, 0.9$), capture ($p = 0.2, 0.5, 0.9$), and tag retention ($\lambda = 0.2, 0.5, 0.9$) probabilities. The black line indicates the true value of p used to simulate the data for each model.

Web Table 6: Standard Error (SE), Standard Deviation (SD), and Root Mean Squared Error (RMSE) of capture estimates ($\hat{p}$) of 100 simulated datasets analyzed excluding (R') and including(R) the effect of recycled individuals with population size $N = 100000$ with $T_2 = 1$ with 10 sample times for varying tag retention ($\lambda = 0.2, 0.5, 0.9$), varying survival probabilities ($\phi = 0.2, 0.5, 0.9$) and varying capture probabilities ($p = 0.2, 0.5, 0.9$).

ϕ	p	λ	SE (R')	SE (R)	SD (R')	SD (R)	RMSE (R')	RMSE (R)
0.9	0.9	0.9	0.0007	0.0006	0.0007	0.0007	0.0007	0.0007
0.9	0.9	0.5	0.0014	0.0011	0.0013	0.0011	0.0013	0.0011
0.9	0.9	0.2	0.0039	0.0025	0.0035	0.0023	0.0035	0.0023
0.9	0.5	0.9	0.0013	0.0013	0.0012	0.0012	0.0012	0.0012
0.9	0.5	0.5	0.0025	0.0020	0.0125	0.0090	0.0176	0.0228
0.9	0.5	0.2	0.0062	0.0045	0.0062	0.0045	0.0171	0.0172
0.9	0.2	0.9	0.0015	0.0015	0.0018	0.0016	0.0084	0.0091
0.9	0.2	0.5	0.0021	0.0020	0.0033	0.0020	0.0188	0.0147
0.9	0.2	0.2	0.0041	0.0032	0.0038	0.0028	0.0361	0.0441
0.5	0.9	0.9	0.0017	0.0017	0.0017	0.0017	0.0016	0.0017
0.5	0.9	0.5	0.0029	0.0026	0.0029	0.0027	0.0028	0.0027
0.5	0.9	0.2	0.0098	0.0089	0.0426	0.0084	0.0782	0.0925
0.5	0.5	0.9	0.0032	0.0032	0.0033	0.0033	0.0033	0.0033
0.5	0.5	0.5	0.0054	0.0051	0.0059	0.0057	0.0059	0.0057
0.5	0.5	0.2	0.0075	0.0074	0.0539	0.0433	0.1715	0.1502
0.5	0.2	0.9	0.0025	0.0022	0.0739	0.0781	0.0892	0.1011
0.5	0.2	0.5	0.0053	0.0052	0.0249	0.0220	0.0260	0.0228
0.5	0.2	0.2	0.0045	0.0043	0.0038	0.0035	0.0863	0.0871
0.2	0.9	0.9	0.0050	0.0050	0.0043	0.0796	0.0043	0.0801
0.2	0.9	0.5	0.0077	0.0075	0.0078	0.0078	0.0078	0.0077
0.2	0.9	0.2	0.0160	0.0151	0.0149	0.0136	0.4861	0.4862
0.2	0.5	0.9	0.0092	0.0092	0.0098	0.0098	0.0097	0.0097
0.2	0.5	0.5	0.0125	0.0118	0.1248	0.1411	0.1340	0.1561
0.2	0.5	0.2	0.0053	0.0051	0.0067	0.0068	0.3540	0.3544
0.2	0.2	0.9	0.0093	0.0092	0.0092	0.0092	0.0092	0.0092
0.2	0.2	0.5	0.0021	0.0021	0.0020	0.0019	0.1478	0.1479
0.2	0.2	0.2	0.0046	0.0045	0.0040	0.0040	0.1402	0.1403

N=1000, T=0.5

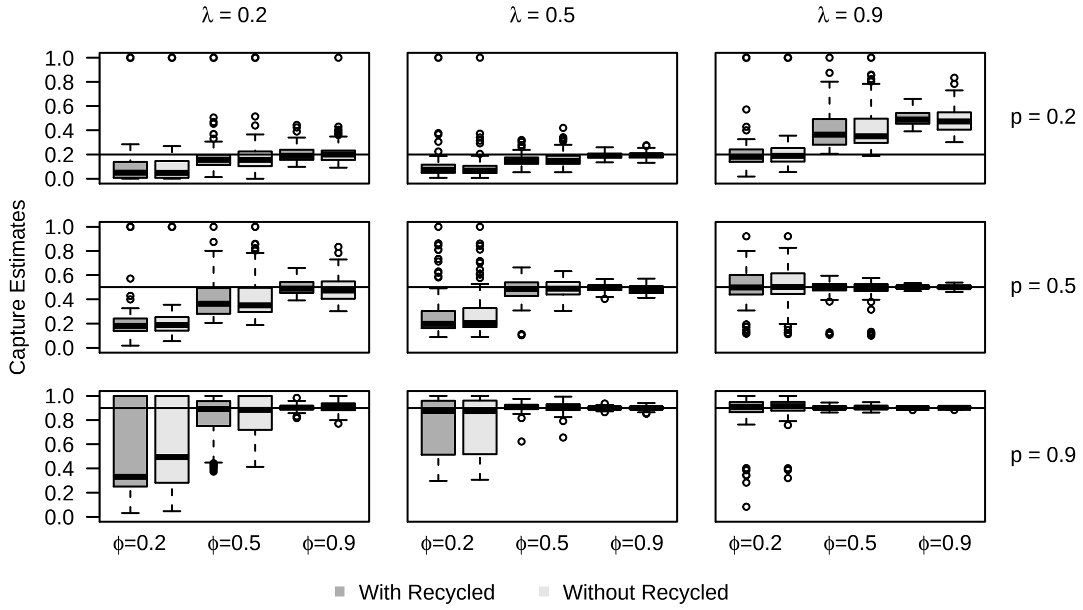

Web Figure 9: Boxplots of capture estimates ($\hat{p}$) of 100 simulated datasets analyzed including and excluding the effect of recycled individuals for population size 1000 with $T_2 = 0.5$ with 10 sample times for varying survival ($\phi = 0.2, 0.5, 0.9$), capture ($p = 0.2, 0.5, 0.9$), and tag retention ($\lambda = 0.2, 0.5, 0.9$) probabilities. The black line indicates the true value of p used to simulate the data for each model.

Web Table 7: Standard Error (SE), Standard Deviation (SD), and Root Mean Squared Error (RMSE) of capture estimates ($\hat{p}$) of 100 simulated datasets analyzed excluding (R') and including(R) the effect of recycled individuals with population size $N = 1000$ with $T_2 = 0.5$ with 10 sample times for varying tag retention ($\lambda = 0.2, 0.5, 0.9$), varying survival probabilities ($\phi = 0.2, 0.5, 0.9$) and varying capture probabilities ($p = 0.2, 0.5, 0.9$).

ϕ	p	λ	SE (R')	SE (R)	SD (R')	SD (R)	RMSE (R')	RMSE (R)
0.9	0.9	0.9	0.0070	0.0066	0.0065	0.0063	0.0066	0.0063
0.9	0.9	0.5	0.0159	0.0115	0.0171	0.0118	0.0170	0.0118
0.9	0.9	0.2	0.0412	0.0269	0.0432	0.0272	0.0438	0.0271
0.9	0.5	0.9	0.0139	0.0134	0.0151	0.0140	0.0150	0.0140
0.9	0.5	0.5	0.0279	0.0239	0.0363	0.0290	0.0408	0.0291
0.9	0.5	0.2	0.0688	0.0549	0.1051	0.0602	0.1064	0.0599
0.9	0.2	0.9	0.0156	0.0152	0.0179	0.0170	0.0181	0.0175
0.9	0.2	0.5	0.0276	0.0247	0.0280	0.0244	0.0287	0.0256
0.9	0.2	0.2	0.0674	0.0622	0.1086	0.0699	0.1091	0.0696
0.5	0.9	0.9	0.0176	0.0173	0.0183	0.0178	0.0184	0.0179
0.5	0.9	0.5	0.0311	0.0277	0.0407	0.0397	0.0407	0.0398
0.5	0.9	0.2	0.0638	0.0612	0.2004	0.1960	0.2177	0.2129
0.5	0.5	0.9	0.0320	0.0321	0.1063	0.1001	0.1092	0.1015
0.5	0.5	0.5	0.0574	0.0520	0.0813	0.1080	0.0826	0.1120
0.5	0.5	0.2	0.1038	0.0960	0.1778	0.1638	0.1954	0.1866
0.5	0.2	0.9	0.0324	0.0318	0.0592	0.0613	0.0618	0.0646
0.5	0.2	0.5	0.0432	0.0399	0.0640	0.0555	0.0744	0.0710
0.5	0.2	0.2	0.0811	0.0812	0.2551	0.2047	0.2573	0.2037
0.2	0.9	0.9	0.0444	0.0437	0.1088	0.1639	0.1083	0.1654
0.2	0.9	0.5	0.0733	0.0723	0.2443	0.2453	0.2780	0.2848
0.2	0.9	0.2	0.0924	0.0772	0.3353	0.3400	0.4540	0.5007
0.2	0.5	0.9	0.0952	0.0935	0.1384	0.1450	0.1390	0.1445
0.2	0.5	0.5	0.0725	0.0707	0.1847	0.1933	0.2873	0.2928
0.2	0.5	0.2	0.0641	0.0659	0.2176	0.2122	0.3340	0.3404
0.2	0.2	0.9	0.0502	0.0511	0.1418	0.1057	0.1607	0.1355
0.2	0.2	0.5	0.0496	0.0484	0.1133	0.1435	0.1544	0.1723
0.2	0.2	0.2	0.0437	0.0381	0.2166	0.2775	0.2291	0.2801

N=100000, T=0.5

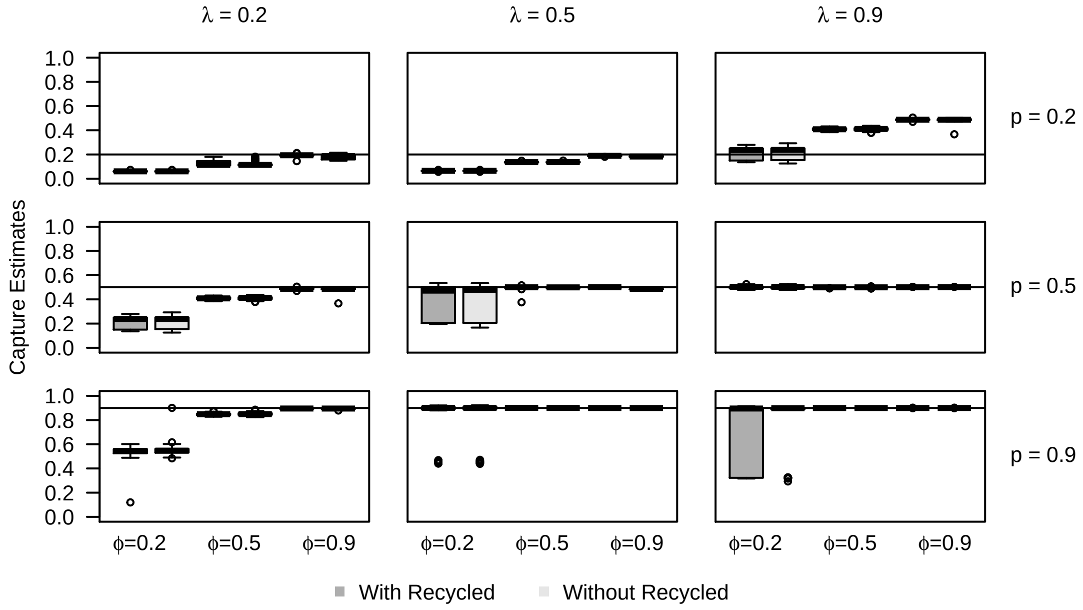

Web Figure 10: Boxplots of capture estimates ($\hat{p}$) of 100 simulated datasets analyzed including and excluding the effect of recycled individuals for population size 100000 with $T_2 = 0.5$ with 10 sample times for varying survival ($\phi = 0.2, 0.5, 0.9$), capture ($p = 0.2, 0.5, 0.9$), and tag retention ($\lambda = 0.2, 0.5, 0.9$) probabilities. The black line indicates the true value of p used to simulate the data for each model.

Web Table 8: Standard Error (SE), Standard Deviation (SD), and Root Mean Squared Error (RMSE) of capture estimates ($\hat{p}$) of 100 simulated datasets analyzed excluding (R') and including(R) the effect of recycled individuals with population size $N = 100000$ with $T_2 = 0.5$ with 10 sample times for varying tag retention ($\lambda = 0.2, 0.5, 0.9$), varying survival probabilities ($\phi = 0.2, 0.5, 0.9$) and varying capture probabilities ($p = 0.2, 0.5, 0.9$).

ϕ	p	λ	SE (R')	SE (R)	SD (R')	SD (R)	RMSE (R')	RMSE (R)
0.9	0.9	0.9	0.0007	0.0007	0.0007	0.0007	0.0007	0.0007
0.9	0.9	0.5	0.0016	0.0011	0.0016	0.0011	0.0016	0.0011
0.9	0.9	0.2	0.0046	0.0028	0.0048	0.0032	0.0068	0.0058
0.9	0.5	0.9	0.0014	0.0013	0.0013	0.0013	0.0013	0.0013
0.9	0.5	0.5	0.0028	0.0024	0.0028	0.0024	0.0161	0.0024
0.9	0.5	0.2	0.0074	0.0053	0.0141	0.0052	0.0193	0.0131
0.9	0.2	0.9	0.0015	0.0016	0.0032	0.0033	0.0092	0.0035
0.9	0.2	0.5	0.0025	0.0024	0.0050	0.0026	0.0166	0.0113
0.9	0.2	0.2	0.0060	0.0059	0.0195	0.0077	0.0283	0.0101
0.5	0.9	0.9	0.0018	0.0017	0.0018	0.0018	0.0018	0.0018
0.5	0.9	0.5	0.0032	0.0029	0.0029	0.0029	0.0029	0.0030
0.5	0.9	0.2	0.0112	0.0092	0.0114	0.0097	0.0518	0.0529
0.5	0.5	0.9	0.0034	0.0033	0.0032	0.0031	0.0032	0.0031
0.5	0.5	0.5	0.0061	0.0056	0.0061	0.0138	0.0061	0.0138
0.5	0.5	0.2	0.0121	0.0106	0.0109	0.0102	0.0908	0.0918
0.5	0.2	0.9	0.0034	0.0034	0.0261	0.0204	0.0281	0.0212
0.5	0.2	0.5	0.0036	0.0035	0.0036	0.0035	0.0638	0.0640
0.5	0.2	0.2	0.0061	0.0061	0.0188	0.0212	0.0815	0.0788
0.2	0.9	0.9	0.0048	0.0045	0.2409	0.2749	0.2716	0.3372
0.2	0.9	0.5	0.0088	0.0086	0.1520	0.1609	0.1613	0.1730
0.2	0.9	0.2	0.0279	0.0259	0.0436	0.0488	0.3536	0.3631
0.2	0.5	0.9	0.0096	0.0096	0.0097	0.0097	0.0097	0.0097
0.2	0.5	0.5	0.0116	0.0109	0.1466	0.1493	0.1926	0.2039
0.2	0.5	0.2	0.0134	0.0126	0.0485	0.0469	0.2877	0.2907
0.2	0.2	0.9	0.0081	0.0078	0.0616	0.0651	0.0680	0.0733
0.2	0.2	0.5	0.0032	0.0032	0.0028	0.0028	0.1350	0.1350
0.2	0.2	0.2	0.0054	0.0053	0.0043	0.0042	0.1393	0.1396

N=1000, T=1, t=5

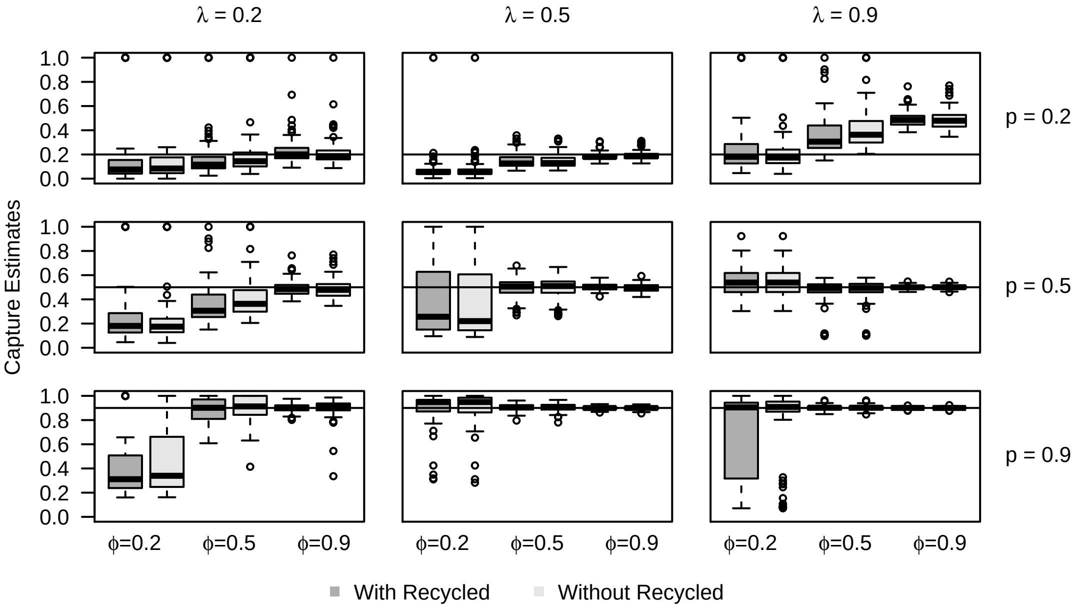

WEB FIGURE 11: Boxplots of capture estimates ($\hat{p}$) of 100 simulated datasets analyzed including and excluding the effect of recycled individuals for population size 1000 with $T_2 = 1$ for 5 sample times for varying survival ($\phi = 0.2, 0.5, 0.9$), capture ($p = 0.2, 0.5, 0.9$), and tag retention ($\lambda = 0.2, 0.5, 0.9$) probabilities. The black line indicates the true value of p used to simulate the data for each model.

N=1000, T=1, t=7

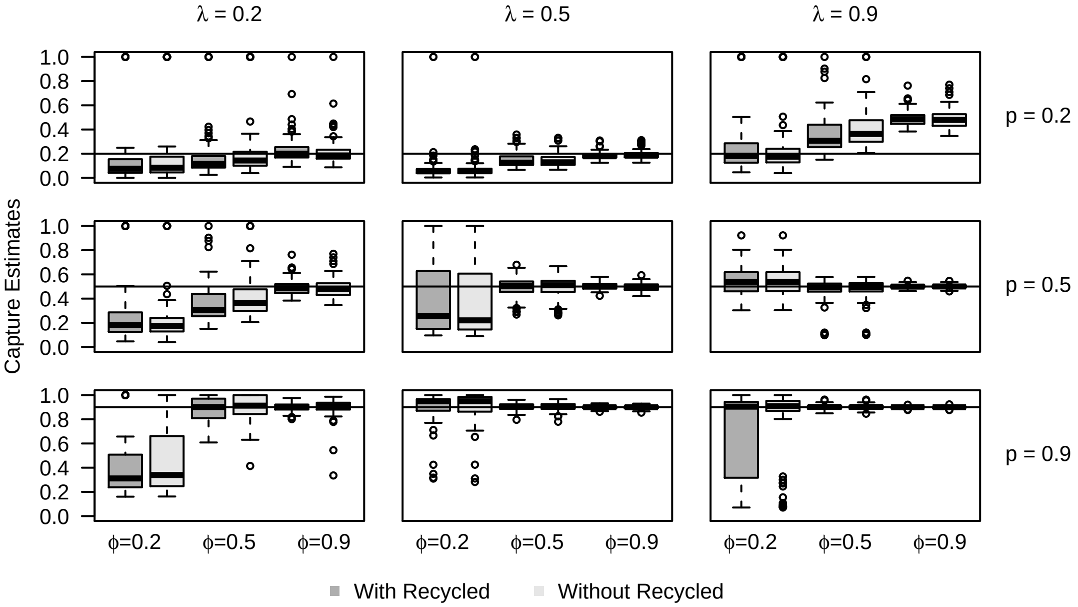

WEB FIGURE 12: Boxplots of capture estimates ($\hat{p}$) of 100 simulated datasets analyzed including and excluding the effect of recycled individuals for population size 1000 with $T_2 = 1$ for 7 sample times for varying survival ($\phi = 0.2, 0.5, 0.9$), capture ($p = 0.2, 0.5, 0.9$), and tag retention ($\lambda = 0.2, 0.5, 0.9$) probabilities. The black line indicates the true value of p used to simulate the data for each model.

Tag Retention Estimates

N=1000, T=1

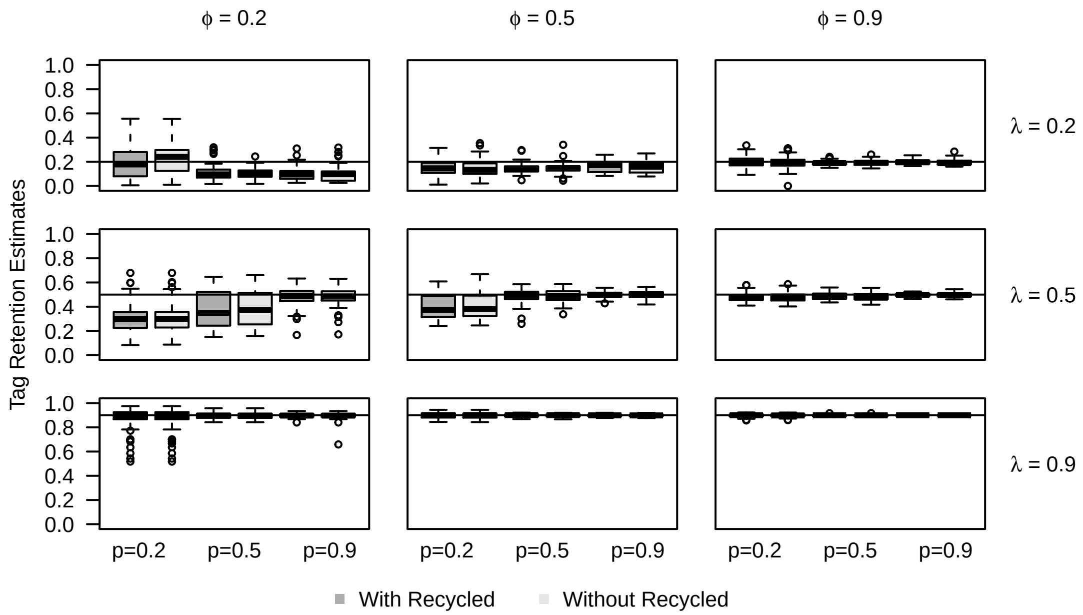

WEB FIGURE 13: Boxplots of tag retention estimates ($\hat{\lambda}$) of 100 simulated datasets analyzed including and excluding the effect of recycled individuals for population size 1000 with $T_2 = 1$ with 10 sample times for varying survival ($\phi = 0.2, 0.5, 0.9$), capture ($p = 0.2, 0.5, 0.9$), and tag retention ($\lambda = 0.2, 0.5, 0.9$) probabilities. The black line indicates the true value of λ used to simulate the data for each model.

WEB TABLE 9: Standard Error (SE), Standard Deviation (SD), and Root Mean Squared Error (RMSE) of tag retention estimates ($\hat{\lambda}$) of 100 simulated datasets analyzed without (R') and with (R) the effect of recycled individuals with population size $N = 1000$ with $T_2 = 1$ with 10 sample times for varying tag retention ($\lambda = 0.2, 0.5, 0.9$), varying survival probabilities ($\phi = 0.2, 0.5, 0.9$) and varying capture probabilities ($p = 0.2, 0.5, 0.9$).

ϕ	p	λ	SE (R')	SE (R)	SD (R')	SD (R)	RMSE (R')	RMSE (R)
0.9	0.9	0.9	0.0051	0.0049	0.0054	0.0054	0.0054	0.0054
0.9	0.9	0.5	0.0197	0.0143	0.0188	0.0142	0.0191	0.0144
0.9	0.9	0.2	0.0234	0.0152	0.0239	0.0184	0.0242	0.0184
0.9	0.5	0.9	0.0065	0.0063	0.0065	0.0061	0.0064	0.0061
0.9	0.5	0.5	0.0207	0.0184	0.0324	0.0291	0.0367	0.0314
0.9	0.5	0.2	0.0203	0.0178	0.0226	0.0187	0.0243	0.0210
0.9	0.2	0.9	0.0114	0.0113	0.0127	0.0129	0.0127	0.0130
0.9	0.2	0.5	0.0326	0.0297	0.0408	0.0349	0.0469	0.0417
0.9	0.2	0.2	0.0431	0.0386	0.0463	0.0439	0.0462	0.0437
0.5	0.9	0.9	0.0093	0.0092	0.0091	0.0091	0.0091	0.0091
0.5	0.9	0.5	0.0283	0.0256	0.0294	0.0273	0.0294	0.0272
0.5	0.9	0.2	0.0254	0.0260	0.0495	0.0478	0.0655	0.0597
0.5	0.5	0.9	0.0123	0.0122	0.0116	0.0115	0.0115	0.0115
0.5	0.5	0.5	0.0389	0.0363	0.0489	0.0511	0.0497	0.0517
0.5	0.5	0.2	0.0267	0.0250	0.0370	0.0371	0.0652	0.0680
0.5	0.2	0.9	0.0224	0.0223	0.0229	0.0224	0.0228	0.0223
0.5	0.2	0.5	0.0566	0.0551	0.1072	0.1040	0.1410	0.1421
0.5	0.2	0.2	0.0514	0.0480	0.0653	0.0597	0.0838	0.0784
0.2	0.9	0.9	0.0171	0.0170	0.0292	0.0165	0.0294	0.0165
0.2	0.9	0.5	0.0468	0.0457	0.0692	0.0673	0.0711	0.0686
0.2	0.9	0.2	0.0219	0.0234	0.0539	0.0521	0.1168	0.1139
0.2	0.5	0.9	0.0240	0.0240	0.0222	0.0220	0.0224	0.0222
0.2	0.5	0.5	0.0541	0.0526	0.1386	0.1432	0.1791	0.1866
0.2	0.5	0.2	0.0325	0.0323	0.0405	0.0639	0.1047	0.1109
0.2	0.2	0.9	0.0513	0.0517	0.0858	0.0857	0.0884	0.0885
0.2	0.2	0.5	0.0898	0.0899	0.1087	0.1069	0.2187	0.2229
0.2	0.2	0.2	0.0889	0.0824	0.1151	0.1192	0.1159	0.1194

N=100000, T=1

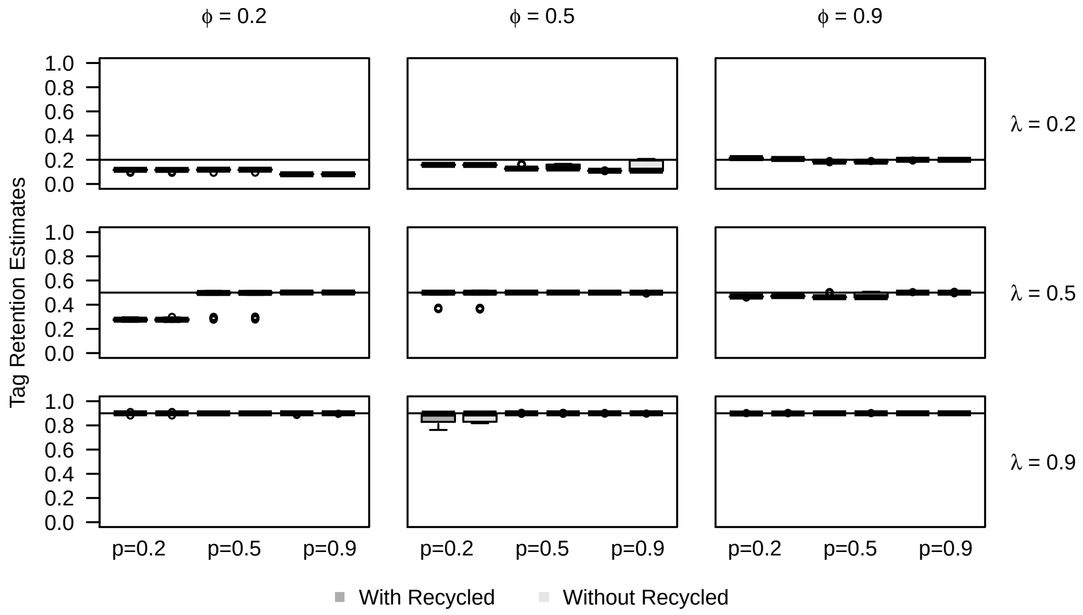

Web Figure 14: Boxplots of tag retention estimates ($\hat{\lambda}$) of 100 simulated datasets analyzed including and excluding the effect of recycled individuals for population size 100000 with $T_2 = 1$ with 10 sample times for varying survival ($\phi = 0.2, 0.5, 0.9$), capture ($p = 0.2, 0.5, 0.9$), and tag retention ($\lambda = 0.2, 0.5, 0.9$) probabilities. The black line indicates the true value of λ used to simulate the data for each model.

Web Table 10: Standard Error (SE), Standard Deviation (SD), and Root Mean Squared Error (RMSE) of tag retention estimates ($\hat{\lambda}$) of 100 simulated datasets analyzed without (R') and with (R) the effect of recycled individuals with population size $N = 1000$ with $T_2 = 1$ with 10 sample times for varying tag retention ($\lambda = 0.2, 0.5, 0.9$), varying survival probabilities ($\phi = 0.2, 0.5, 0.9$) and varying capture probabilities ($p = 0.2, 0.5, 0.9$).

ϕ	p	λ	SE (R')	SE (R)	SD (R')	SD (R)	RMSE (R')	RMSE (R)
0.9	0.9	0.9	0.0051	0.0049	0.0054	0.0054	0.0054	0.0054
0.9	0.9	0.5	0.0197	0.0143	0.0188	0.0142	0.0191	0.0144
0.9	0.9	0.2	0.0234	0.0152	0.0239	0.0184	0.0242	0.0184
0.9	0.5	0.9	0.0065	0.0063	0.0065	0.0061	0.0064	0.0061
0.9	0.5	0.5	0.0207	0.0184	0.0324	0.0291	0.0367	0.0314
0.9	0.5	0.2	0.0203	0.0178	0.0226	0.0187	0.0243	0.0210
0.9	0.2	0.9	0.0114	0.0113	0.0127	0.0129	0.0127	0.0130
0.9	0.2	0.5	0.0326	0.0297	0.0408	0.0349	0.0469	0.0417
0.9	0.2	0.2	0.0431	0.0386	0.0463	0.0439	0.0462	0.0437
0.5	0.9	0.9	0.0093	0.0092	0.0091	0.0091	0.0091	0.0091
0.5	0.9	0.5	0.0283	0.0256	0.0294	0.0273	0.0294	0.0272
0.5	0.9	0.2	0.0254	0.0260	0.0495	0.0478	0.0655	0.0597
0.5	0.5	0.9	0.0123	0.0122	0.0116	0.0115	0.0115	0.0115
0.5	0.5	0.5	0.0389	0.0363	0.0489	0.0511	0.0497	0.0517
0.5	0.5	0.2	0.0267	0.0250	0.0370	0.0371	0.0652	0.0680
0.5	0.2	0.9	0.0224	0.0223	0.0229	0.0224	0.0228	0.0223
0.5	0.2	0.5	0.0566	0.0551	0.1072	0.1040	0.1410	0.1421
0.5	0.2	0.2	0.0514	0.0480	0.0653	0.0597	0.0838	0.0784
0.2	0.9	0.9	0.0171	0.0170	0.0292	0.0165	0.0294	0.0165
0.2	0.9	0.5	0.0468	0.0457	0.0692	0.0673	0.0711	0.0686
0.2	0.9	0.2	0.0219	0.0234	0.0539	0.0521	0.1168	0.1139
0.2	0.5	0.9	0.0240	0.0240	0.0222	0.0220	0.0224	0.0222
0.2	0.5	0.5	0.0541	0.0526	0.1386	0.1432	0.1791	0.1866
0.2	0.5	0.2	0.0325	0.0323	0.0405	0.0639	0.1047	0.1109
0.2	0.2	0.9	0.0513	0.0517	0.0858	0.0857	0.0884	0.0885
0.2	0.2	0.5	0.0898	0.0899	0.1087	0.1069	0.2187	0.2229
0.2	0.2	0.2	0.0889	0.0824	0.1151	0.1192	0.1159	0.1194

N=1000, T=0.5

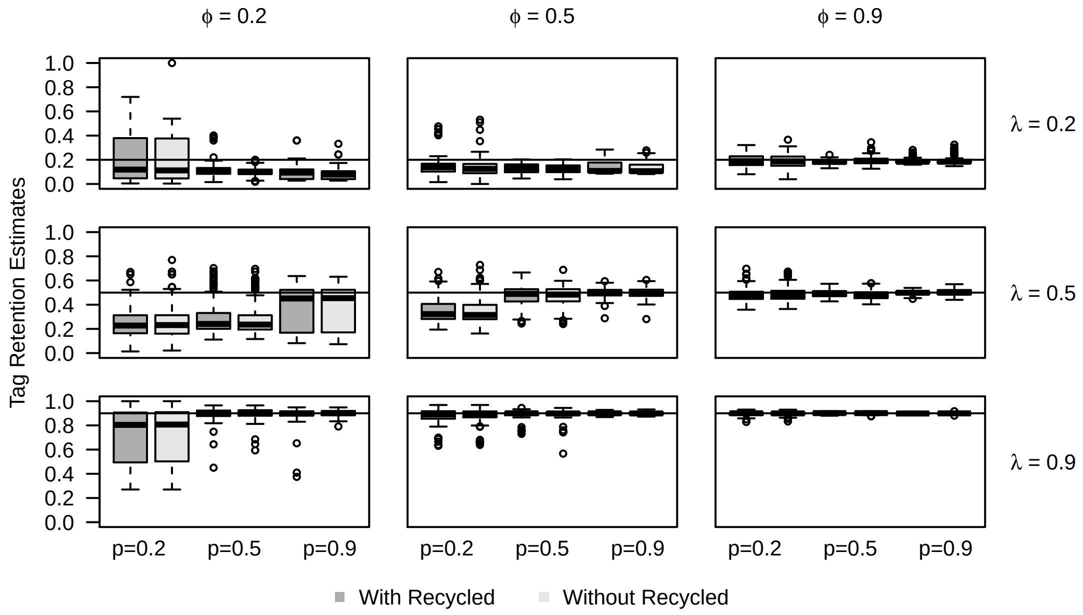

Web Figure 15: Boxplots of tag retention estimates ($\hat{\lambda}$) of 100 simulated datasets analyzed including and excluding the effect of recycled individuals for population size 1000 with $T_2 = 0.5$ with 10 sample times for varying survival ($\phi = 0.2, 0.5, 0.9$), capture ($p = 0.2, 0.5, 0.9$), and tag retention ($\lambda = 0.2, 0.5, 0.9$) probabilities. The black line indicates the true value of λ used to simulate the data for each model.

Web Table 11: Standard Error (SE), Standard Deviation (SD), and Root Mean Squared Error (RMSE) of tag retention estimates ($\hat{\lambda}$) of 100 simulated datasets analyzed without (R') and with (R) the effect of recycled individuals with population size $N = 1000$ with $T_2 = 0.5$ with 10 sample times for varying tag retention ($\lambda = 0.2, 0.5, 0.9$), varying survival probabilities ($\phi = 0.2, 0.5, 0.9$) and varying capture probabilities ($p = 0.2, 0.5, 0.9$).

ϕ	p	λ	SE (R')	SE (R)	SD (R')	SD (R)	RMSE (R')	RMSE (R)
0.9	0.9	0.9	0.0069	0.0064	0.0067	0.0062	0.0067	0.0063
0.9	0.9	0.5	0.0268	0.0185	0.0262	0.0178	0.0262	0.0179
0.9	0.9	0.2	0.0186	0.0118	0.0346	0.0229	0.0352	0.0247
0.9	0.5	0.9	0.0087	0.0083	0.0081	0.0084	0.0080	0.0084
0.9	0.5	0.5	0.0242	0.0256	0.0392	0.0282	0.0429	0.0288
0.9	0.5	0.2	0.0253	0.0177	0.0345	0.0192	0.0350	0.0270
0.9	0.2	0.9	0.0157	0.0156	0.0168	0.0172	0.0169	0.0174
0.9	0.2	0.5	0.0421	0.0370	0.0602	0.0584	0.0617	0.0604
0.9	0.2	0.2	0.0498	0.0452	0.0538	0.0492	0.0547	0.0499
0.5	0.9	0.9	0.0129	0.0126	0.0130	0.0128	0.0130	0.0128
0.5	0.9	0.5	0.0387	0.0342	0.0446	0.0420	0.0445	0.0418
0.5	0.9	0.2	0.0170	0.0172	0.0432	0.0508	0.0867	0.0835
0.5	0.5	0.9	0.0172	0.0170	0.0519	0.0379	0.0532	0.0387
0.5	0.5	0.5	0.0510	0.0435	0.0865	0.0964	0.0922	0.1040
0.5	0.5	0.2	0.0277	0.0253	0.0372	0.0385	0.0839	0.0812
0.5	0.2	0.9	0.0340	0.0340	0.0696	0.0659	0.0731	0.0699
0.5	0.2	0.5	0.0627	0.0623	0.1117	0.1002	0.1838	0.1766
0.5	0.2	0.2	0.0563	0.0597	0.0879	0.0909	0.1079	0.1024
0.2	0.9	0.9	0.0240	0.0241	0.0269	0.0792	0.0268	0.0801
0.2	0.9	0.5	0.0510	0.0477	0.1798	0.1830	0.2166	0.2237
0.2	0.9	0.2	0.0252	0.0219	0.0497	0.0564	0.1273	0.1229
0.2	0.5	0.9	0.0340	0.0341	0.0554	0.0625	0.0555	0.0629
0.2	0.5	0.5	0.0545	0.0537	0.1572	0.1479	0.2558	0.2533
0.2	0.5	0.2	0.0346	0.0360	0.0376	0.0723	0.1056	0.1104
0.2	0.2	0.9	0.0897	0.0907	0.2184	0.2189	0.2911	0.2890
0.2	0.2	0.5	0.0930	0.0911	0.1403	0.1252	0.2771	0.2813
0.2	0.2	0.2	0.0855	0.0907	0.1852	0.1774	0.1843	0.1766

N=100000, T=0.5

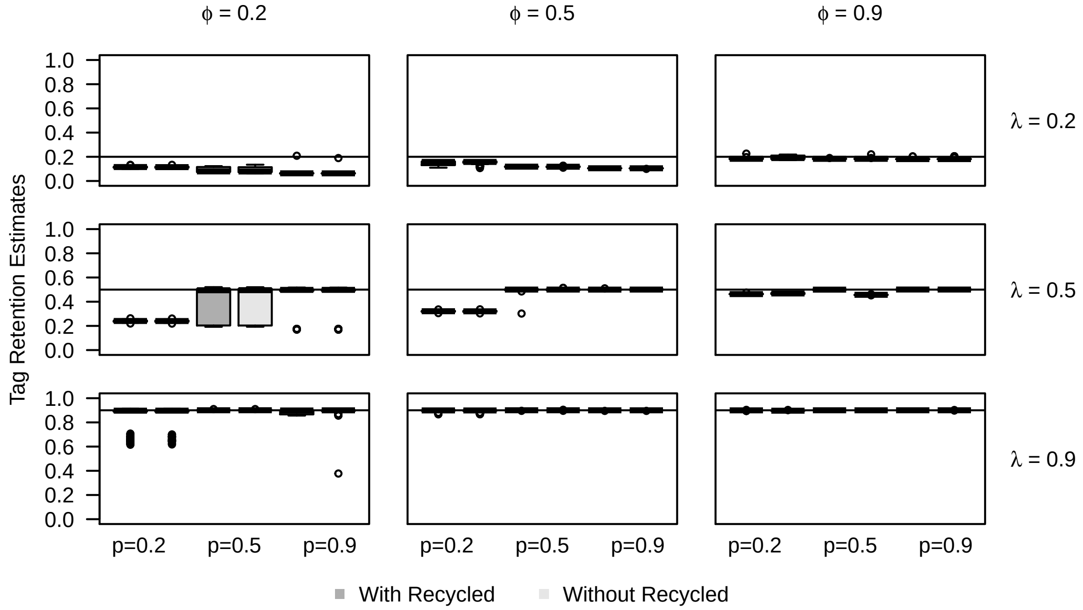

Web Figure 16: Boxplots of tag retention estimates ($\hat{\lambda}$) of 100 simulated datasets analyzed including and excluding the effect of recycled individuals for population size 100000 with $T_2 = 0.5$ with 10 sample times for varying survival ($\phi = 0.2, 0.5, 0.9$), capture ($p = 0.2, 0.5, 0.9$), and tag retention ($\lambda = 0.2, 0.5, 0.9$) probabilities. The black line indicates the true value of λ used to simulate the data for each model.

Web Table 12: Standard Error (SE), Standard Deviation (SD), and Root Mean Squared Error (RMSE) of tag retention estimates ($\hat{\lambda}$) of 100 simulated datasets analyzed without (R') and with (R) the effect of recycled individuals with population size $N = 100000$ with $T_2 = 0.5$ with 10 sample times for varying tag retention ($\lambda = 0.2, 0.5, 0.9$), varying survival probabilities ($\phi = 0.2, 0.5, 0.9$) and varying capture probabilities ($p = 0.2, 0.5, 0.9$).

ϕ	p	λ	SE (R')	SE (R)	SD (R')	SD (R)	RMSE (R')	RMSE (R)
0.9	0.9	0.9	0.0007	0.0006	0.0007	0.0006	0.0007	0.0006
0.9	0.9	0.5	0.0027	0.0019	0.0028	0.0019	0.0028	0.0020
0.9	0.9	0.2	0.0016	0.0008	0.0064	0.0033	0.0177	0.0190
0.9	0.5	0.9	0.0009	0.0008	0.0008	0.0008	0.0008	0.0008
0.9	0.5	0.5	0.0017	0.0029	0.0018	0.0026	0.0431	0.0026
0.9	0.5	0.2	0.0024	0.0017	0.0045	0.0017	0.0171	0.0169
0.9	0.2	0.9	0.0016	0.0015	0.0019	0.0018	0.0035	0.0019
0.9	0.2	0.5	0.0036	0.0033	0.0054	0.0036	0.0330	0.0370
0.9	0.2	0.2	0.0052	0.0046	0.0148	0.0061	0.0155	0.0166
0.5	0.9	0.9	0.0013	0.0013	0.0013	0.0013	0.0013	0.0013
0.5	0.9	0.5	0.0039	0.0034	0.0038	0.0033	0.0038	0.0033
0.5	0.9	0.2	0.0014	0.0012	0.0014	0.0011	0.0951	0.0950
0.5	0.5	0.9	0.0017	0.0017	0.0016	0.0015	0.0016	0.0015
0.5	0.5	0.5	0.0054	0.0050	0.0053	0.0207	0.0053	0.0207
0.5	0.5	0.2	0.0031	0.0027	0.0029	0.0027	0.0819	0.0816
0.5	0.2	0.9	0.0032	0.0031	0.0093	0.0079	0.0101	0.0082
0.5	0.2	0.5	0.0056	0.0055	0.0053	0.0051	0.1794	0.1790
0.5	0.2	0.2	0.0064	0.0061	0.0151	0.0175	0.0504	0.0536
0.2	0.9	0.9	0.0026	0.0027	0.0537	0.0174	0.0550	0.0212
0.2	0.9	0.5	0.0060	0.0058	0.1104	0.1171	0.1182	0.1269
0.2	0.9	0.2	0.0031	0.0028	0.0129	0.0147	0.1360	0.1359
0.2	0.5	0.9	0.0033	0.0033	0.0032	0.0032	0.0032	0.0032
0.2	0.5	0.5	0.0072	0.0068	0.1502	0.1518	0.1932	0.2040
0.2	0.5	0.2	0.0046	0.0044	0.0200	0.0192	0.1130	0.1120
0.2	0.2	0.9	0.0080	0.0082	0.0922	0.0985	0.1018	0.1107
0.2	0.2	0.5	0.0090	0.0089	0.0075	0.0075	0.2603	0.2601
0.2	0.2	0.2	0.0092	0.0091	0.0070	0.0070	0.0854	0.0850

N=1000, T=1, t=5

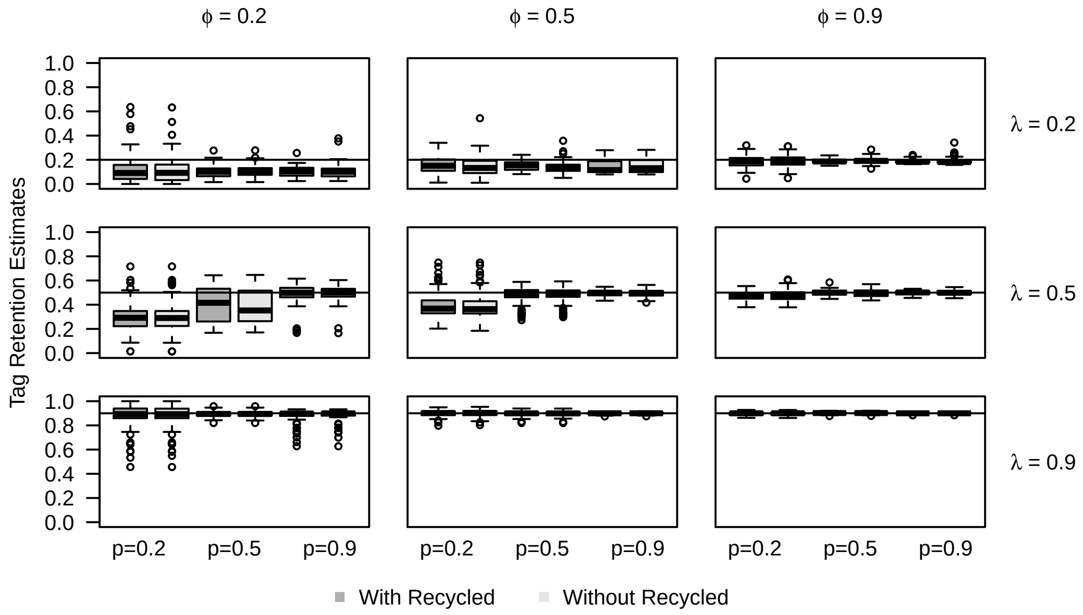

Web Figure 17: Boxplots of tag retention estimates ($\hat{\lambda}$) of 100 simulated datasets analyzed including and excluding the effect of recycled individuals for population size 1000 with $T_2 = 1$ for 5 sample times for varying survival ($\phi = 0.2, 0.5, 0.9$), capture ($p = 0.2, 0.5, 0.9$), and tag retention ($\lambda = 0.2, 0.5, 0.9$) probabilities. The black line indicates the true value of λ used to simulate the data for each model.

N=1000, T=1, t=7

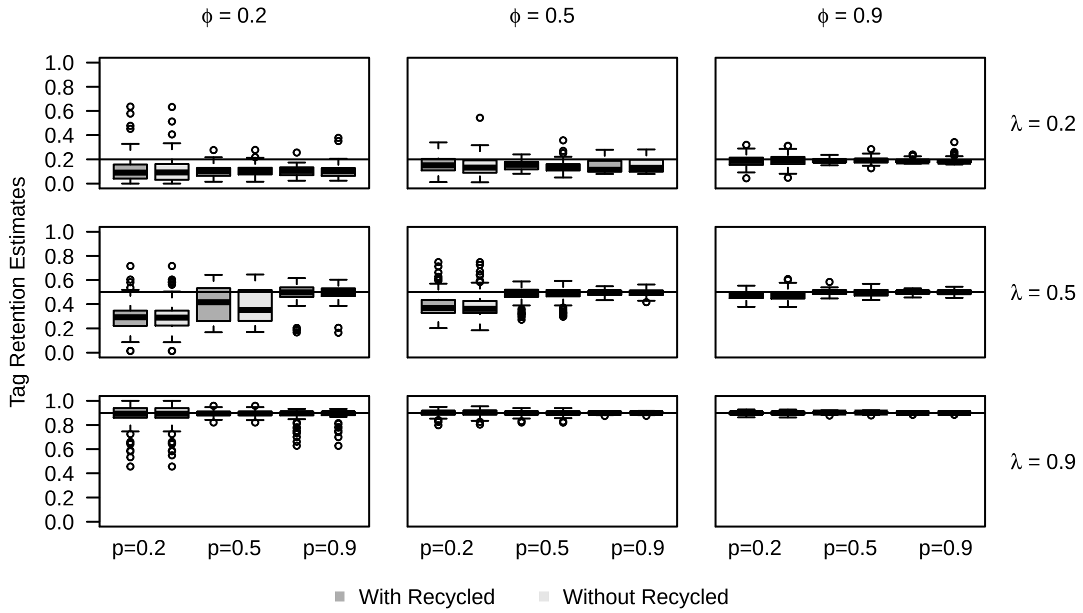

Web Figure 18: Boxplots of tag retention estimates ($\hat{\lambda}$) of 100 simulated datasets analyzed including and excluding the effect of recycled individuals for population size 1000 with $T_2 = 1$ for 7 sample times for varying survival ($\phi = 0.2, 0.5, 0.9$), capture ($p = 0.2, 0.5, 0.9$), and tag retention ($\lambda = 0.2, 0.5, 0.9$) probabilities. The black line indicates the true value of λ used to simulate the data for each model.

Super-Population Size Estimates

N=1000, T=1

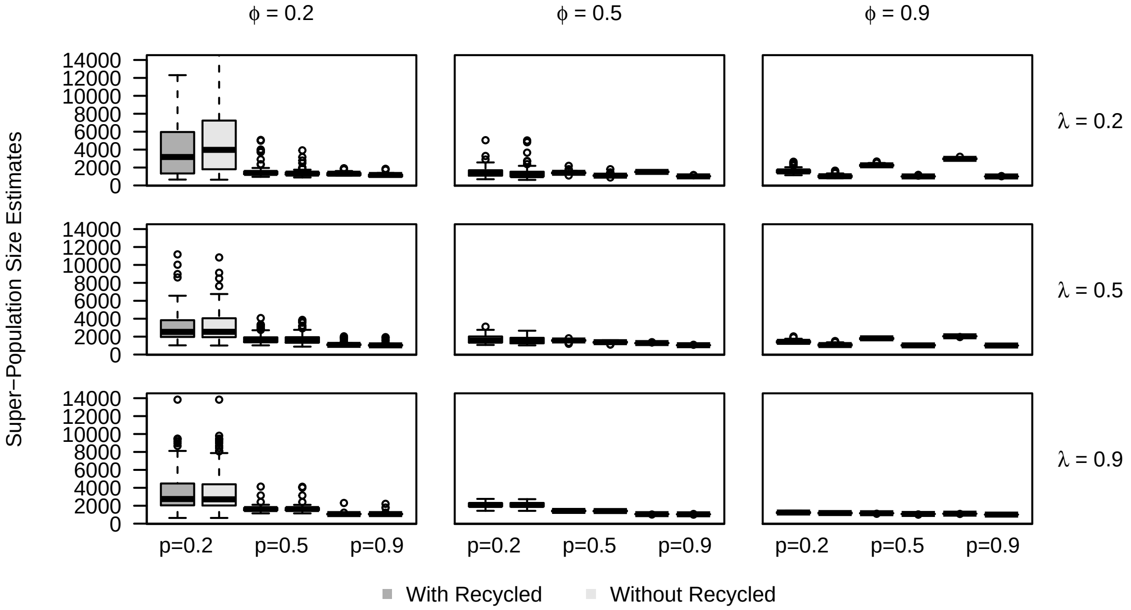

Web Figure 19: Boxplots of super-population size estimates (N) of 100 simulated datasets analyzed including and excluding the effect of recycled individuals for population size 1000 with $T_2 = 1$ with 10 sample times for varying survival ($\phi = 0.2, 0.5, 0.9$), capture ($p = 0.2, 0.5, 0.9$), and tag retention ($\lambda = 0.2, 0.5, 0.9$) probabilities. The black line indicates the true value of N used to simulate the data for each model.

Web Table 13: Standard Error (SE), Standard Deviation (SD), and Root Mean Squared Error (RMSE) of super-population size estimates (N) of 100 simulated datasets analyzed excluding (R') and including(R) the effect of recycled individuals with population size $N = 1000$ with $T_2 = 1$ with 10 sample times for varying tag retention ($\lambda = 0.2, 0.5, 0.9$), varying survival probabilities ($\phi = 0.2, 0.5, 0.9$) and varying capture probabilities ($p = 0.2, 0.5, 0.9$).

ϕ	p	λ	SE (R')	SE (R)	SD (R')	SD (R)	RMSE (R')	RMSE (R)
0.9	0.9	0.9	50.5	50.5	4.0	11.1	10.8	114.5
0.9	0.9	0.5	50.5	50.5	5.8	33.0	11.0	1037.9
0.9	0.9	0.2	49.6	49.6	10.5	75.8	12.0	1980.5
0.9	0.5	0.9	50.5	50.5	19.0	21.9	80.8	164.5
0.9	0.5	0.5	50.5	50.5	54.5	82.5	68.2	801.9
0.9	0.5	0.2	50.5	50.5	34.9	112.9	37.0	1266.4
0.9	0.2	0.9	49.8	49.8	74.2	80.3	192.9	245.4
0.9	0.2	0.5	50.5	50.5	130.0	160.7	156.0	477.6
0.9	0.2	0.2	49.6	50.5	173.7	290.1	179.3	687.8
0.5	0.9	0.9	50.5	50.5	12.0	12.7	49.1	64.7
0.5	0.9	0.5	50.5	50.5	16.9	28.4	48.0	284.1
0.5	0.9	0.2	49.6	49.6	34.7	57.0	44.2	527.3
0.5	0.5	0.9	50.5	50.5	61.6	61.6	411.9	424.5
0.5	0.5	0.5	50.4	50.4	85.0	95.9	388.2	576.5
0.5	0.5	0.2	50.5	50.5	108.7	132.5	157.8	443.4
0.5	0.2	0.9	49.4	49.4	276.4	273.7	1152.3	1149.4
0.5	0.2	0.5	50.5	50.5	417.9	445.8	723.5	816.6
0.5	0.2	0.2	49.4	49.4	710.4	587.7	801.9	760.4
0.2	0.9	0.9	50.5	50.5	158.2	132.9	184.1	156.7
0.2	0.9	0.5	50.8	50.5	176.7	165.7	194.4	214.2
0.2	0.9	0.2	50.3	50.3	161.5	164.5	226.2	346.0
0.2	0.5	0.9	50.5	50.5	426.7	362.3	815.9	763.0
0.2	0.5	0.5	50.5	50.5	624.0	582.9	973.6	954.5
0.2	0.5	0.2	50.5	50.5	423.4	784.3	583.8	993.3
0.2	0.2	0.9	50.5	50.5	2517.1	2485.1	3651.5	3662.1
0.2	0.2	0.5	50.1	50.1	1852.7	1877.8	2882.2	2866.4
0.2	0.2	0.2	49.1	49.1	8456.1	3760.5	9651.8	5001.5

N=100000, T=0.5

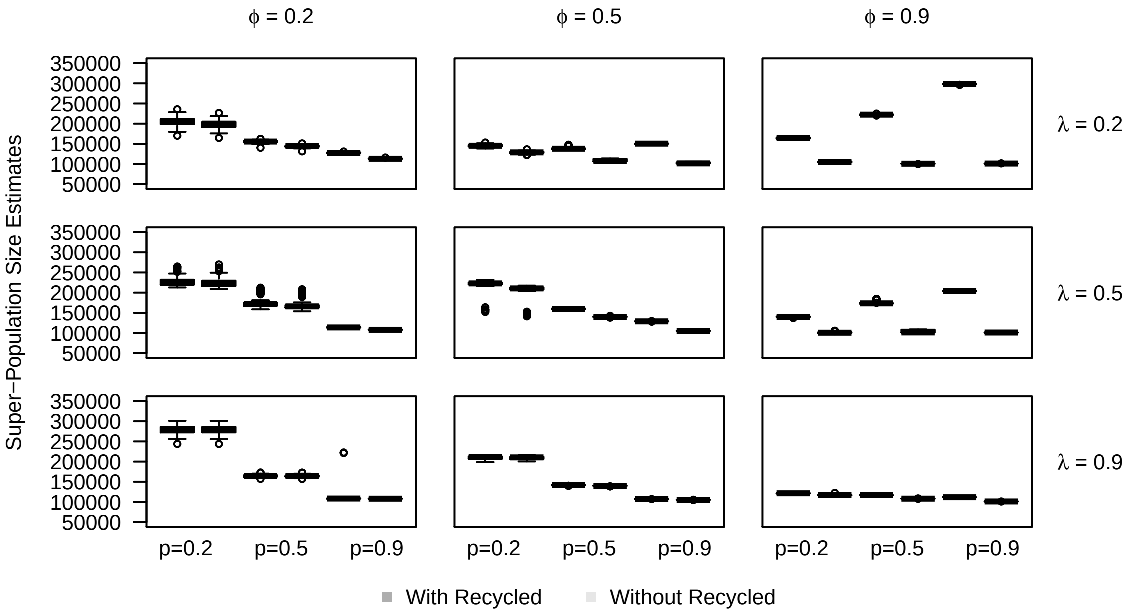

Web Figure 20: Boxplots of super-population size estimates (N) of 100 simulated datasets analyzed including and excluding the effect of recycled individuals for population size 100000 with $T_2 = 1$ with 10 sample times for varying survival ($\phi = 0.2, 0.5, 0.9$), capture ($p = 0.2, 0.5, 0.9$), and tag retention ($\lambda = 0.2, 0.5, 0.9$) probabilities. The black line indicates the true value of N used to simulate the data for each model.

Web Table 14: Standard Error (SE), Standard Deviation (SD), and Root Mean Squared Error (RMSE) of super-population size estimates (N) of 100 simulated datasets analyzed excluding (R') and including(R) the effect of recycled individuals with population size $N = 100000$ with $T_2 = 1$ with 10 sample times for varying tag retention ($\lambda = 0.2, 0.5, 0.9$), varying survival probabilities ($\phi = 0.2, 0.5, 0.9$) and varying capture probabilities ($p = 0.2, 0.5, 0.9$).

ϕ	p	λ	SE (R')	SE (R)	SD (R')	SD (R)	RMSE (R')	RMSE (R)
0.9	0.9	0.9	42.1	42.1	38.9	107.6	988.1	11361.8
0.9	0.9	0.5	37.8	37.8	48.3	355.4	982.7	103591.7
0.9	0.9	0.2	38.5	38.5	135.9	662.0	982.7	198249.4
0.9	0.5	0.9	37.9	37.9	175.4	211.2	7994.0	16488.3
0.9	0.5	0.5	37.9	37.9	3572.1	3521.2	5471.0	74619.3
0.9	0.5	0.2	41.4	41.4	296.0	800.3	712.4	122405.8
0.9	0.2	0.9	49.4	49.4	775.8	564.4	16686.3	21184.5
0.9	0.2	0.5	50.5	50.5	1545.3	993.9	1910.3	40081.3
0.9	0.2	0.2	50.5	50.5	1270.1	1737.6	5261.2	64349.6
0.5	0.9	0.9	50.5	50.5	109.5	117.8	4937.2	6525.9
0.5	0.9	0.5	50.5	50.5	173.4	267.0	4934.9	28655.8
0.5	0.9	0.2	37.3	37.3	1600.4	323.9	2719.0	50378.1
0.5	0.5	0.9	50.5	50.5	595.7	598.0	40018.0	41403.6
0.5	0.5	0.5	37.9	37.9	934.1	1019.7	40127.1	59803.7
0.5	0.5	0.2	38.1	38.1	3118.2	3462.6	9395.9	39297.7
0.5	0.2	0.9	48.9	48.9	3926.0	3936.0	109240.3	109983.6
0.5	0.2	0.5	42.1	42.1	18455.0	17443.4	106660.8	120015.2
0.5	0.2	0.2	50.5	50.5	2571.6	2768.5	28853.1	45396.0
0.2	0.9	0.9	37.9	37.9	428.2	15996.7	7973.9	19083.5
0.2	0.9	0.5	38.3	38.3	772.7	817.7	7954.7	13739.3
0.2	0.9	0.2	38.1	38.1	897.2	969.9	13086.8	27825.1
0.2	0.5	0.9	43.6	43.6	2729.4	2724.0	64215.6	64466.8
0.2	0.5	0.5	38.1	38.1	12500.3	14199.0	70108.9	77289.2
0.2	0.5	0.2	42.0	42.0	2572.1	2739.3	44049.6	55230.3
0.2	0.2	0.9	42.1	42.1	10707.4	10726.6	178913.1	179098.6
0.2	0.2	0.5	50.5	50.5	12705.6	11900.4	125943.1	128430.9
0.2	0.2	0.2	50.5	50.5	9921.4	10271.3	99244.6	105891.3

N=1000, T=0.5

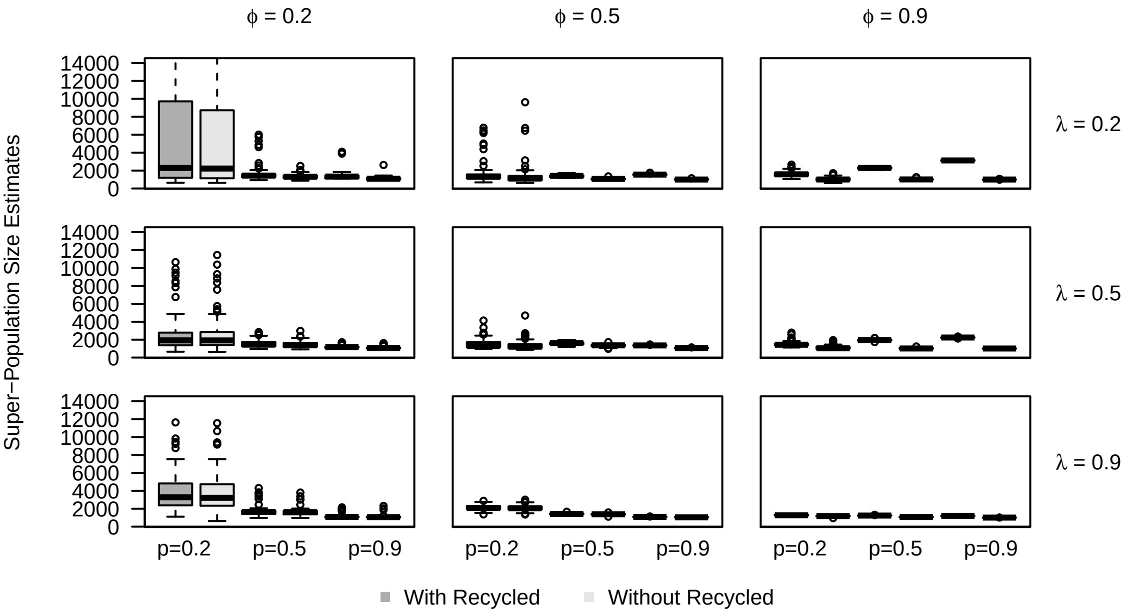

Web Figure 21: Boxplots of super-population size estimates (N) of 100 simulated datasets analyzed including and excluding the effect of recycled individuals for population size 1000 with $T_2 = 0.5$ with 10 sample times for varying survival ($\phi = 0.2, 0.5, 0.9$), capture ($p = 0.2, 0.5, 0.9$), and tag retention ($\lambda = 0.2, 0.5, 0.9$) probabilities. The black line indicates the true value of N used to simulate the data for each model.

Web Table 15: Standard Error (SE), Standard Deviation (SD), and Root Mean Squared Error (RMSE) of super-population size estimates (N) of 100 simulated datasets analyzed excluding (R') and including(R) the effect of recycled individuals with population size $N = 1000$ with $T_2 = 0.5$ with 10 sample times for varying tag retention ($\lambda = 0.2, 0.5, 0.9$), varying survival probabilities ($\phi = 0.2, 0.5, 0.9$) and varying capture probabilities ($p = 0.2, 0.5, 0.9$).

ϕ	p	λ	SE (R')	SE (R)	SD (R')	SD (R)	RMSE (R')	RMSE (R)
0.9	0.9	0.9	50.5	50.5	4.1	13.6	98990.4	98793.7
0.9	0.9	0.5	50.5	50.5	7.1	39.2	98990.7	97760.7
0.9	0.9	0.2	50.5	50.5	13.0	66.0	98996.6	96870.5
0.9	0.5	0.9	50.5	50.5	19.3	28.0	98920.9	98760.1
0.9	0.5	0.5	50.3	50.3	62.8	89.9	98963.0	98058.6
0.9	0.5	0.2	50.5	50.5	54.8	91.8	98984.5	97710.5
0.9	0.2	0.9	50.5	50.5	68.2	75.3	98813.8	98717.0
0.9	0.2	0.5	50.5	50.5	211.3	275.9	98886.7	98472.5
0.9	0.2	0.2	50.5	50.5	197.3	294.3	98961.1	98383.8
0.5	0.9	0.9	50.5	50.5	12.6	13.8	98952.5	98906.8
0.5	0.9	0.5	50.5	50.5	20.8	29.6	98955.0	98645.7
0.5	0.9	0.2	50.4	50.4	35.9	62.7	98981.0	98437.8
0.5	0.5	0.9	50.3	50.3	73.0	70.3	98608.8	98566.8
0.5	0.5	0.5	49.3	49.3	135.1	179.0	98657.1	98440.0
0.5	0.5	0.2	50.5	50.5	89.9	118.4	98932.9	98602.1
0.5	0.2	0.9	50.0	50.0	293.9	276.7	97899.1	97877.0
0.5	0.2	0.5	49.7	49.7	526.1	532.8	98627.1	98483.6
0.5	0.2	0.2	49.4	49.3	1206.1	1205.4	98637.9	98389.6
0.2	0.9	0.9	50.5	50.5	184.9	200.8	98901.6	98873.5
0.2	0.9	0.5	50.3	50.3	130.2	119.9	98908.3	98831.8
0.2	0.9	0.2	49.7	49.7	194.6	410.9	98872.1	98639.7
0.2	0.5	0.9	50.5	50.5	449.9	540.4	98331.1	98283.4
0.2	0.5	0.5	50.2	50.2	357.5	410.7	98531.2	98447.0
0.2	0.5	0.2	50.5	50.5	262.1	903.0	98660.0	98354.8
0.2	0.2	0.9	49.0	49.0	2151.8	2035.6	96164.6	96153.8
0.2	0.2	0.5	48.9	48.9	2042.4	2077.0	97454.8	97469.4
0.2	0.2	0.2	49.1	46.7	11541.5	11170.7	94078.0	93726.1

N=100000, T=0.5

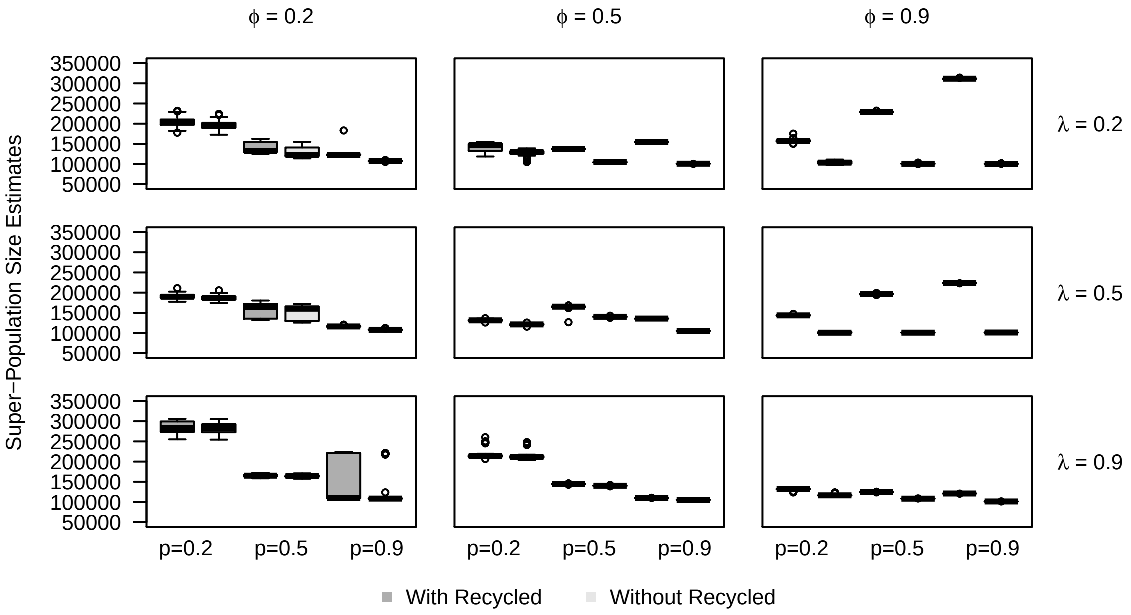

Web Figure 22: Boxplots of super-population size estimates (N) of 100 simulated datasets analyzed including and excluding the effect of recycled individuals for population size 100000 with $T_2 = 0.5$ with 10 sample times for varying survival ($\phi = 0.2, 0.5, 0.9$), capture ($p = 0.2, 0.5, 0.9$), and tag retention ($\lambda = 0.2, 0.5, 0.9$) probabilities. The black line indicates the true value of N used to simulate the data for each model.

Web Table 16: Standard Error (SE), Standard Deviation (SD), and Root Mean Squared Error (RMSE) of super-population size estimates (N) of 100 simulated datasets analyzed excluding (R') and including(R) the effect of recycled individuals with population size $N = 100000$ with $T_2 = 0.5$ with 10 sample times for varying tag retention ($\lambda = 0.2, 0.5, 0.9$), varying survival probabilities ($\phi = 0.2, 0.5, 0.9$) and varying capture probabilities ($p = 0.2, 0.5, 0.9$).

ϕ	p	λ	SE (R')	SE (R)	SD (R')	SD (R)	RMSE (R')	RMSE (R)
0.9	0.9	0.9	50.5	50.5	38.2	154.8	996.5	20737.8
0.9	0.9	0.5	50.5	50.5	65.0	424.6	994.0	124165.7
0.9	0.9	0.2	50.5	50.5	332.4	698.0	384.3	211677.2
0.9	0.5	0.9	50.5	50.5	150.6	230.0	8000.3	24281.5
0.9	0.5	0.5	50.5	50.5	188.6	778.9	716.8	96173.9
0.9	0.5	0.2	50.5	50.5	429.5	836.9	674.7	129334.3
0.9	0.2	0.9	50.5	50.5	1869.8	2206.8	16707.2	31391.7
0.9	0.2	0.5	50.5	50.5	1359.9	1149.9	1675.6	43537.0
0.9	0.2	0.2	50.5	50.5	3394.6	3112.9	5123.0	57428.7
0.5	0.9	0.9	50.5	50.5	120.8	142.7	4955.3	9575.2
0.5	0.9	0.5	50.5	50.5	187.0	302.3	4914.5	35599.2
0.5	0.9	0.2	50.5	50.5	168.8	359.0	679.4	54183.0
0.5	0.5	0.9	50.5	50.5	542.8	560.4	40070.0	44111.9
0.5	0.5	0.5	50.7	50.7	1005.8	4079.9	40103.5	64909.0
0.5	0.5	0.2	50.5	50.5	669.8	911.8	4448.1	37200.1
0.5	0.2	0.9	50.5	50.5	12143.1	10053.7	116273.8	116900.2
0.5	0.2	0.5	50.5	50.5	1854.1	1903.6	21073.9	31154.4
0.5	0.2	0.2	50.5	50.5	7436.9	9739.0	27781.3	43358.6
0.2	0.9	0.9	50.5	50.5	45500.3	53655.2	55134.4	71471.1
0.2	0.9	0.5	49.5	49.5	1354.9	1518.0	8221.2	16562.5
0.2	0.9	0.2	50.5	50.5	863.1	6102.6	7316.1	24173.2
0.2	0.5	0.9	50.5	50.5	2644.7	2650.5	63982.9	65064.7
0.2	0.5	0.5	50.5	50.5	17928.4	18475.8	52981.9	57515.5
0.2	0.5	0.2	50.5	50.5	11448.1	11795.3	30061.5	40722.3
0.2	0.2	0.9	49.5	49.5	102582.5	108004.1	247997.3	257817.0
0.2	0.2	0.5	50.5	50.5	6004.5	6146.6	86974.2	90655.7
0.2	0.2	0.2	47.2	47.2	10566.9	10894.6	97248.1	104776.4

N=1000, T=1, t=5

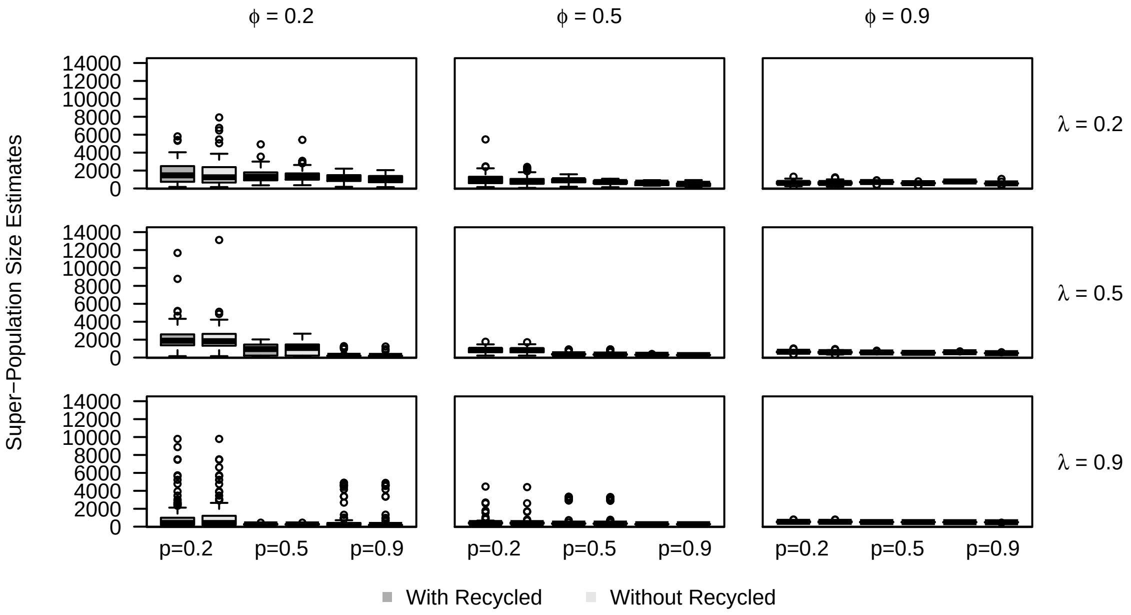

Web Figure 23: Boxplots of super-population size estimates (N) of 100 simulated datasets analyzed including and excluding the effect of recycled individuals for population size 1000 with $T_2 = 1$ for 5 sample times for varying survival ($\phi = 0.2, 0.5, 0.9$), capture ($p = 0.2, 0.5, 0.9$), and tag retention ($\lambda = 0.2, 0.5, 0.9$) probabilities. The black line indicates the true value of N used to simulate the data for each model.

N=1000, T=0.5, t=7

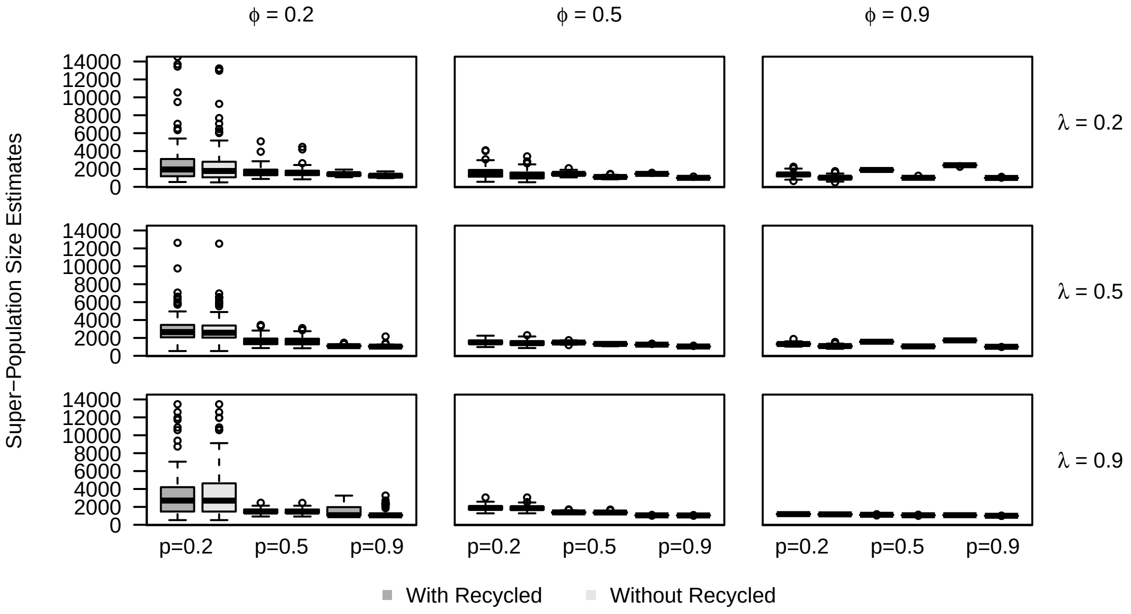

Web Figure 24: Boxplots of super-population size estimates (N) of 100 simulated datasets analyzed including and excluding the effect of recycled individuals for population size 1000 with $T_2 = 1$ for 7 sample times for varying survival ($\phi = 0.2, 0.5, 0.9$), capture ($p = 0.2, 0.5, 0.9$), and tag retention ($\lambda = 0.2, 0.5, 0.9$) probabilities. The black line indicates the true value of N used to simulate the data for each model.

Abundance Estimates

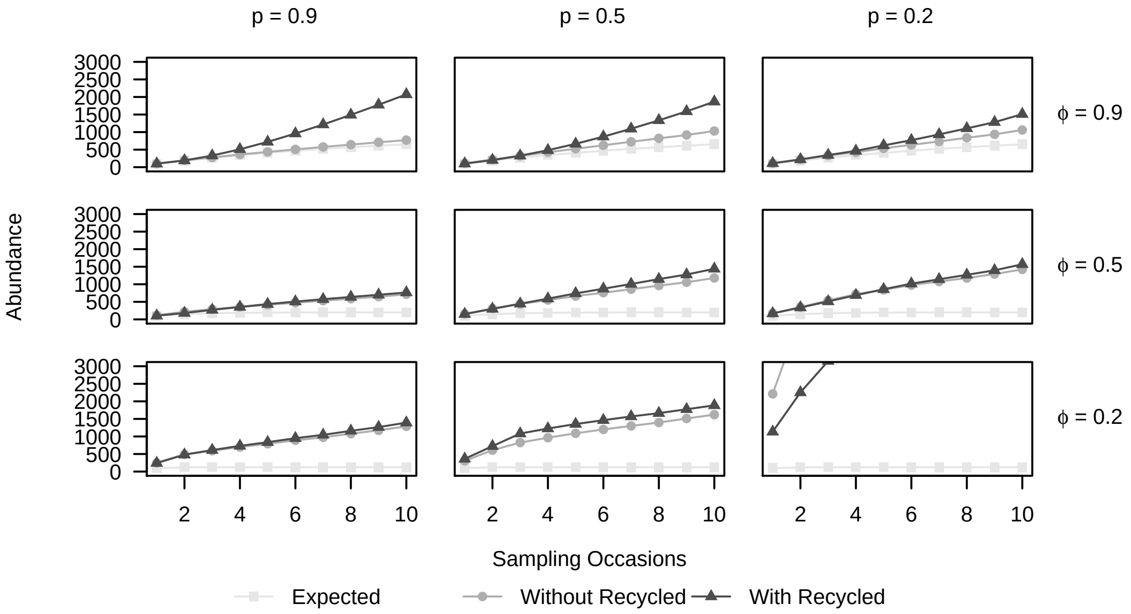

Web Figure 25: Mean abundance estimates (N_j's) for each sample time ($k = 10$) for analysis including and excluding recycled individuals with population size $N = 1000$ with $T_2 = 1$ with 10 sample times for low tag retention ($\lambda = 0.2$), varying survival probabilities ($\phi = 0.2, 0.5, 0.9$) and varying capture probabilities ($p = 0.2, 0.5, 0.9$).

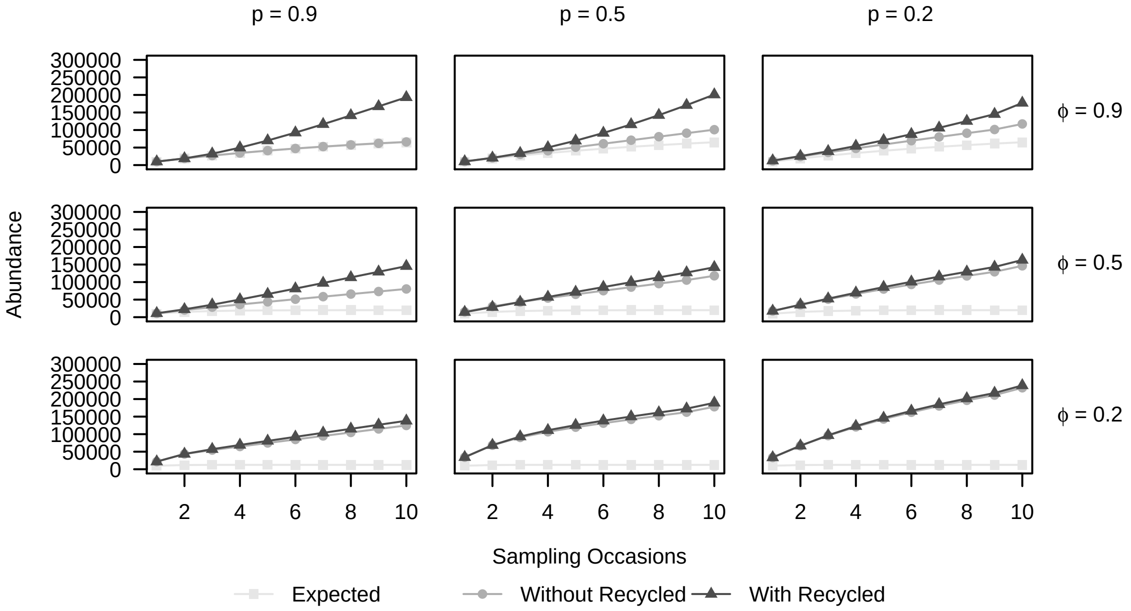

Web Figure 26: Mean abundance estimates (N_j's) for each sample time ($k = 10$) for analysis including and excluding recycled individuals with population size $N = 100000$ with $T_2 = 1$ with 10 sample times for low tag retention ($\lambda = 0.2$), varying survival probabilities ($\phi = 0.2, 0.5, 0.9$) and varying capture probabilities ($p = 0.2, 0.5, 0.9$).

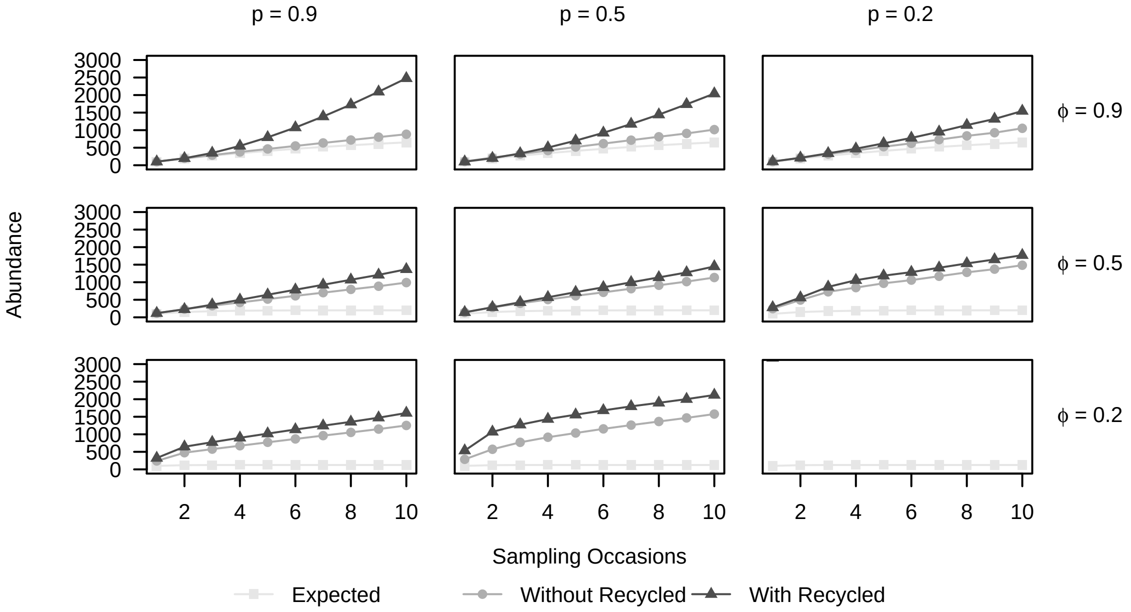

WEB FIGURE 27: Mean abundance estimates (N_j's) for each sample time ($k = 10$) for analysis including and excluding recycled individuals with population size $N = 1000$ with $T_2 = 0.5$ with 10 sample times for low tag retention ($\lambda = 0.2$), varying survival probabilities ($\phi = 0.2, 0.5, 0.9$) and varying capture probabilities ($p = 0.2, 0.5, 0.9$).

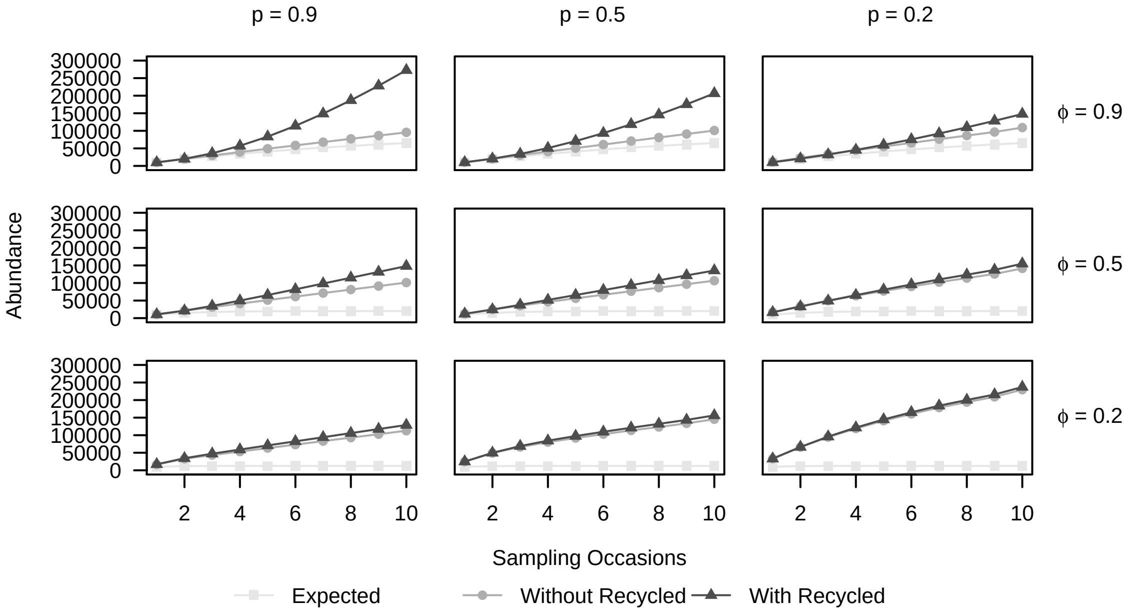

WEB FIGURE 28: Mean abundance estimates (N_j's) for each sample time ($k = 10$) for analysis including and excluding recycled individuals with population size $N = 100000$ with $T_2 = 0.5$ with 10 sample times for low tag retention ($\lambda = 0.2$), varying survival probabilities ($\phi = 0.2, 0.5, 0.9$) and varying capture probabilities ($p = 0.2, 0.5, 0.9$).

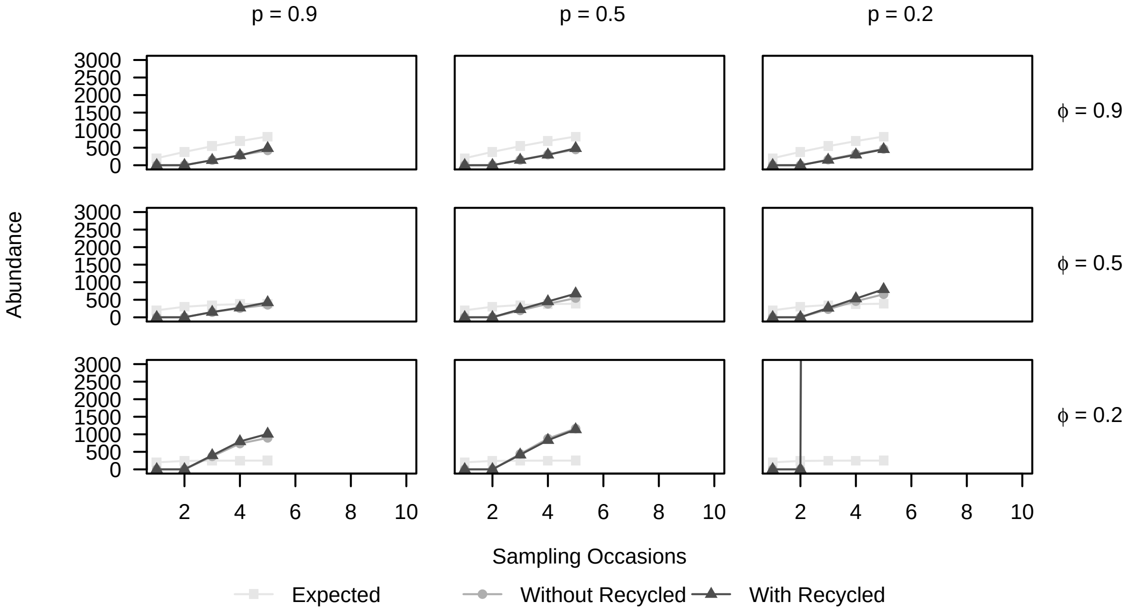

Web Figure 29: Mean abundance estimates (N_j's) for each sample time ($k = 5$) for analysis including and excluding recycled individuals with population size $N = 1000$ with $T_2 = 1$ with 5 sample times for low tag retention ($\lambda = 0.2$), varying survival probabilities ($\phi = 0.2, 0.5, 0.9$) and varying capture probabilities ($p = 0.2, 0.5, 0.9$).

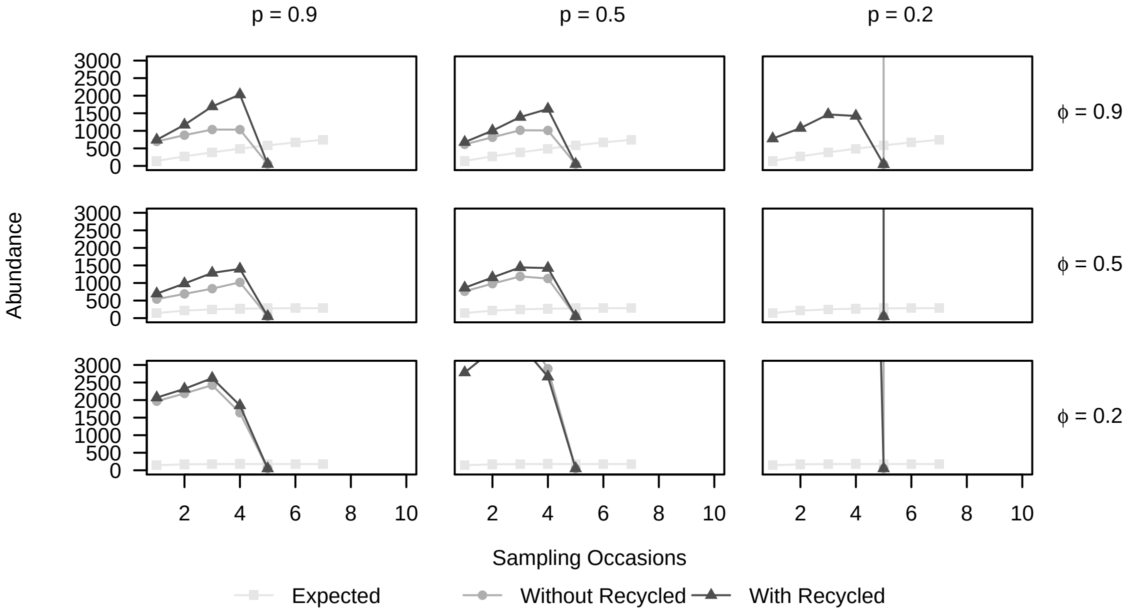

Web Figure 30: Mean abundance estimates (N_j's) for each sample time ($k = 7$) for analysis including and excluding recycled individuals with population size $N = 1000$ with $T_2 = 1$ with 7 sample times for low tag retention ($\lambda = 0.2$), varying survival probabilities ($\phi = 0.2, 0.5, 0.9$) and varying capture probabilities ($p = 0.2, 0.5, 0.9$).

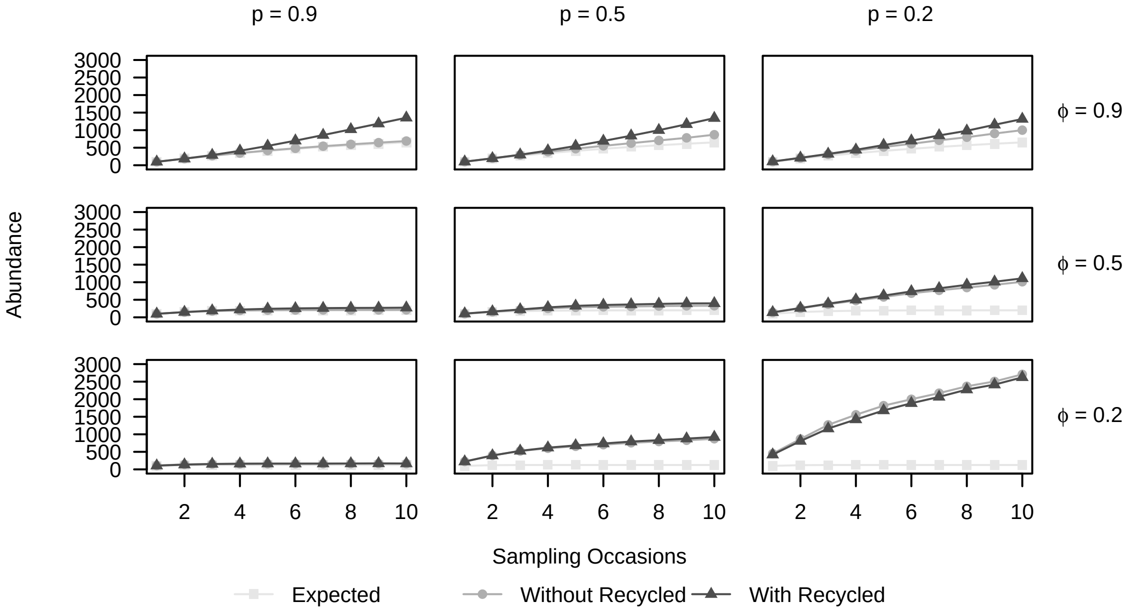

Web Figure 31: Mean abundance estimates (N_j's) for each sample time ($k = 10$) for analysis including and excluding recycled individuals with population size $N = 1000$ with $T_2 = 1$ with 10 sample times for medium tag retention ($\lambda = 0.5$), varying survival probabilities ($\phi = 0.2, 0.5, 0.9$) and varying capture probabilities ($p = 0.2, 0.5, 0.9$).

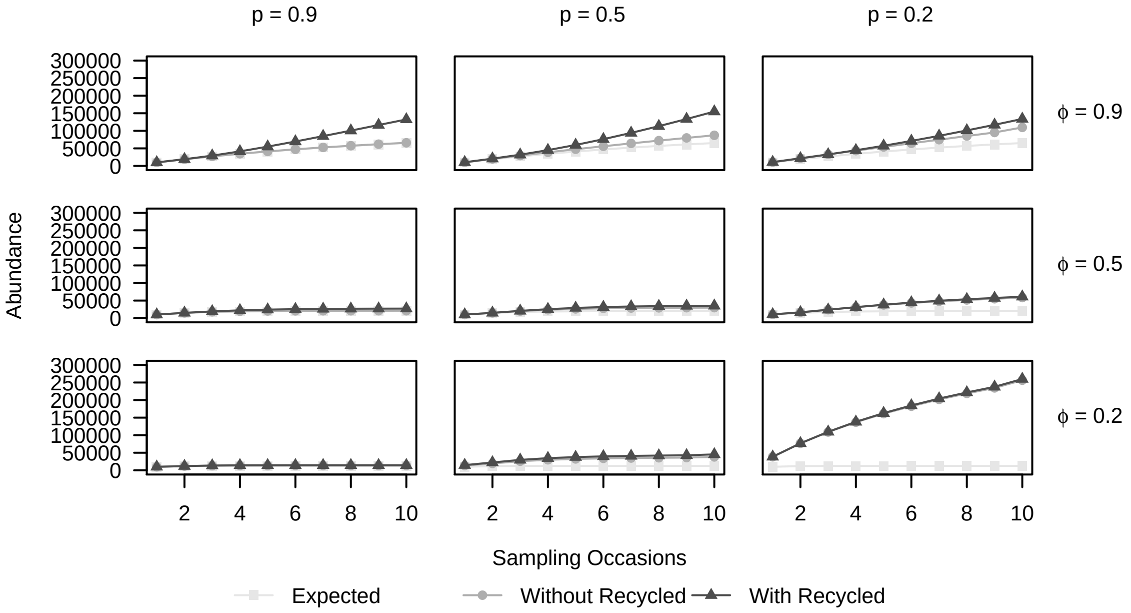

Web Figure 32: Mean abundance estimates (N_j's) for each sample time ($k = 10$) for analysis including and excluding recycled individuals with population size $N = 100000$ with $T_2 = 1$ with 10 sample times for medium tag retention ($\lambda = 0.5$), varying survival probabilities ($\phi = 0.2, 0.5, 0.9$) and varying capture probabilities ($p = 0.2, 0.5, 0.9$).

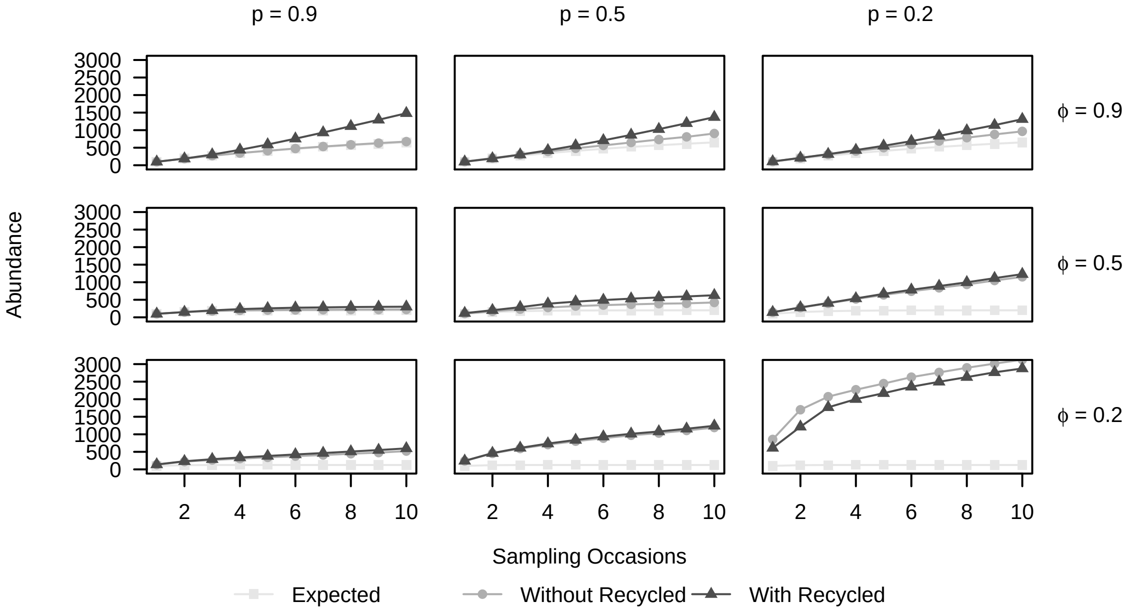

Web Figure 33: Mean abundance estimates (N_j's) for each sample time ($k = 10$) for analysis including and excluding recycled individuals with population size $N = 1000$ with $T_2 = 0.5$ with 10 sample times for medium tag retention ($\lambda = 0.5$), varying survival probabilities ($\phi = 0.2, 0.5, 0.9$) and varying capture probabilities ($p = 0.2, 0.5, 0.9$).

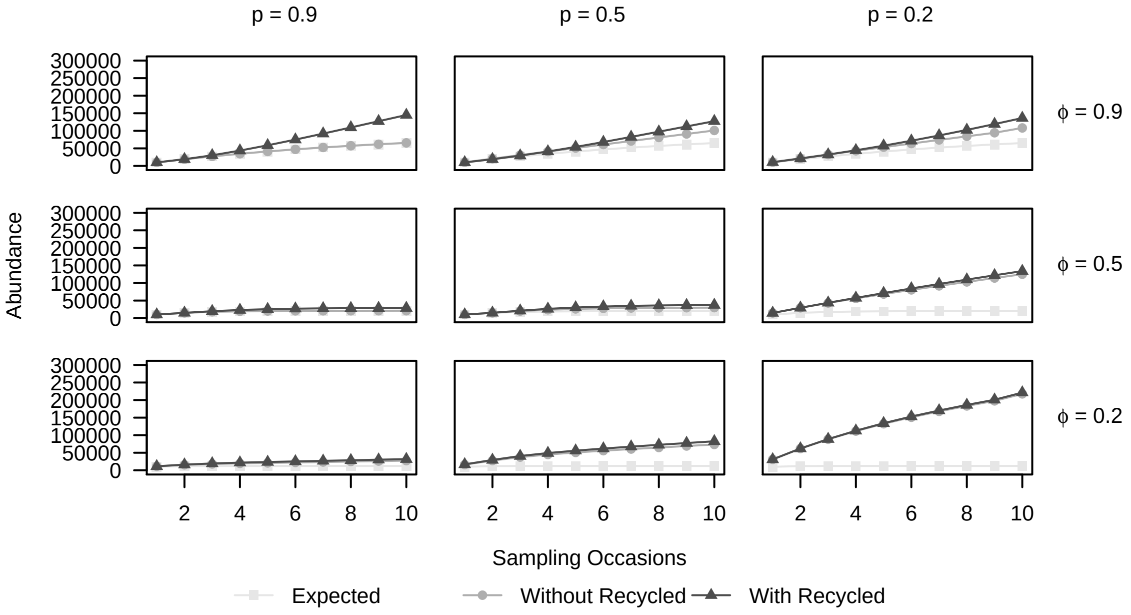

Web Figure 34: Mean abundance estimates (N_j's) for each sample time ($k = 10$) for analysis including and excluding recycled individuals with population size $N = 100000$ with $T_2 = 0.5$ with 10 sample times for medium tag retention ($\lambda = 0.5$), varying survival probabilities ($\phi = 0.2, 0.5, 0.9$) and varying capture probabilities ($p = 0.2, 0.5, 0.9$).

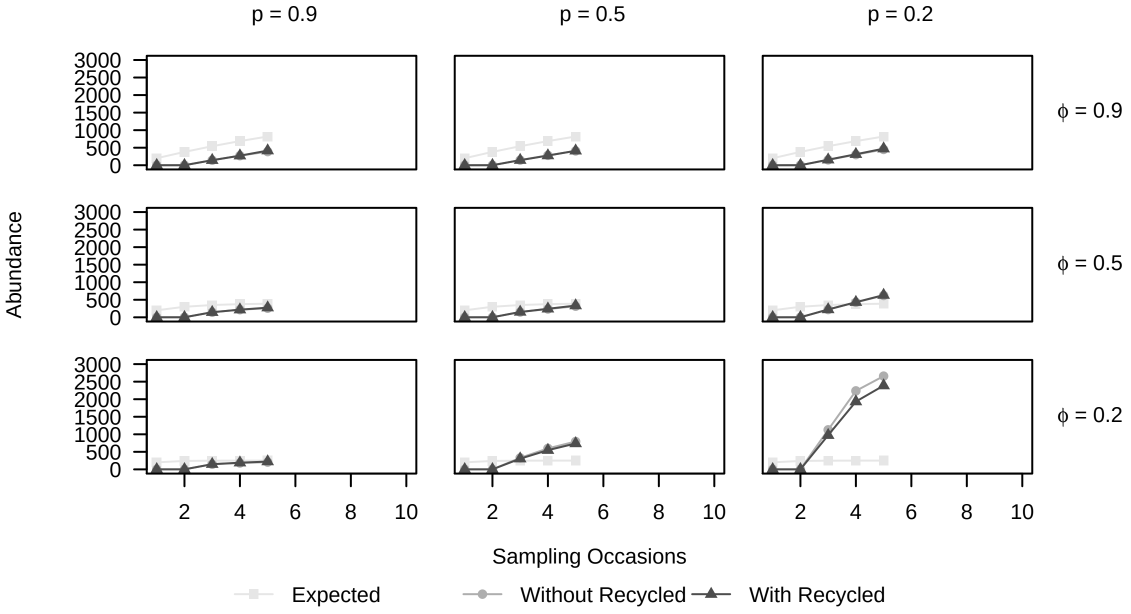

Web Figure 35: Mean abundance estimates (N_j's) for each sample time ($k = 5$) for analysis including and excluding recycled individuals with population size $N = 1000$ with $T_2 = 1$ with 5 sample times for medium tag retention ($\lambda = 0.5$), varying survival probabilities ($\phi = 0.2, 0.5, 0.9$) and varying capture probabilities ($p = 0.2, 0.5, 0.9$).

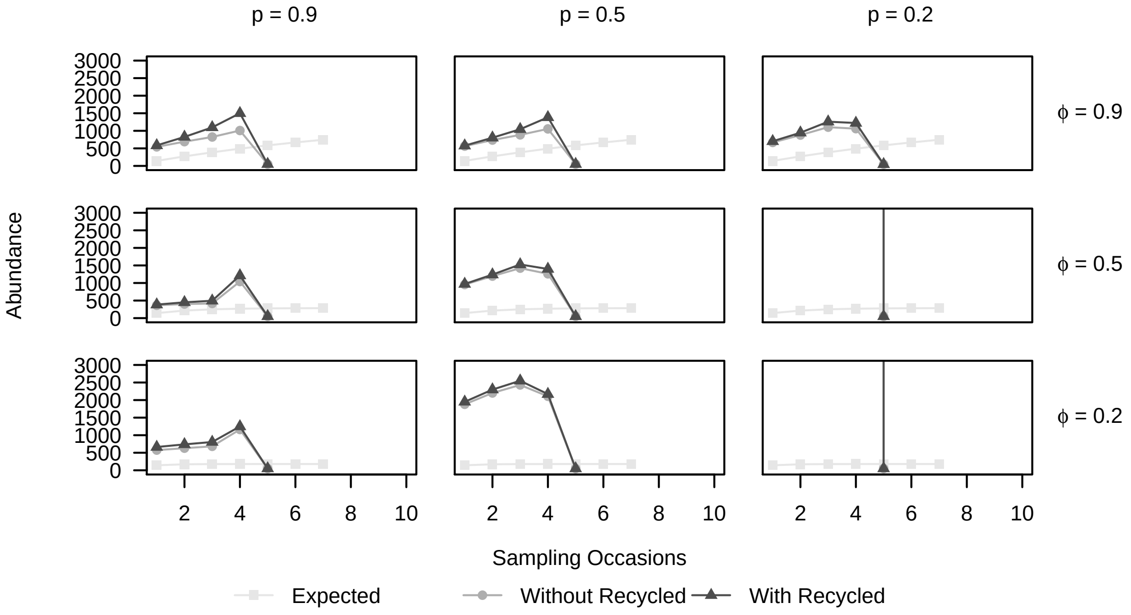

Web Figure 36: Mean abundance estimates (N_j's) for each sample time ($k = 7$) for analysis including and excluding recycled individuals with population size $N = 1000$ with $T_2 = 1$ with 7 sample times for medium tag retention ($\lambda = 0.5$), varying survival probabilities ($\phi = 0.2, 0.5, 0.9$) and varying capture probabilities ($p = 0.2, 0.5, 0.9$).

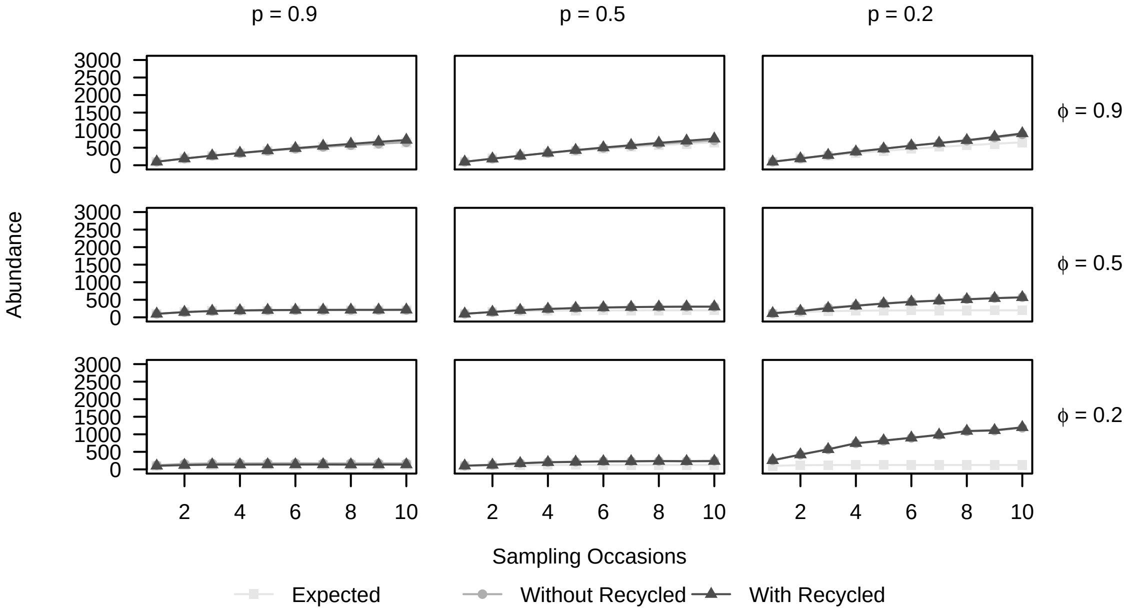

Web Figure 37: Mean abundance estimates (N_j's) for each sample time ($k = 10$) for analysis including and excluding recycled individuals with population size $N = 1000$ with $T_2 = 1$ with 10 sample times for high tag retention ($\lambda = 0.9$), varying survival probabilities ($\phi = 0.2, 0.5, 0.9$) and varying capture probabilities ($p = 0.2, 0.5, 0.9$).

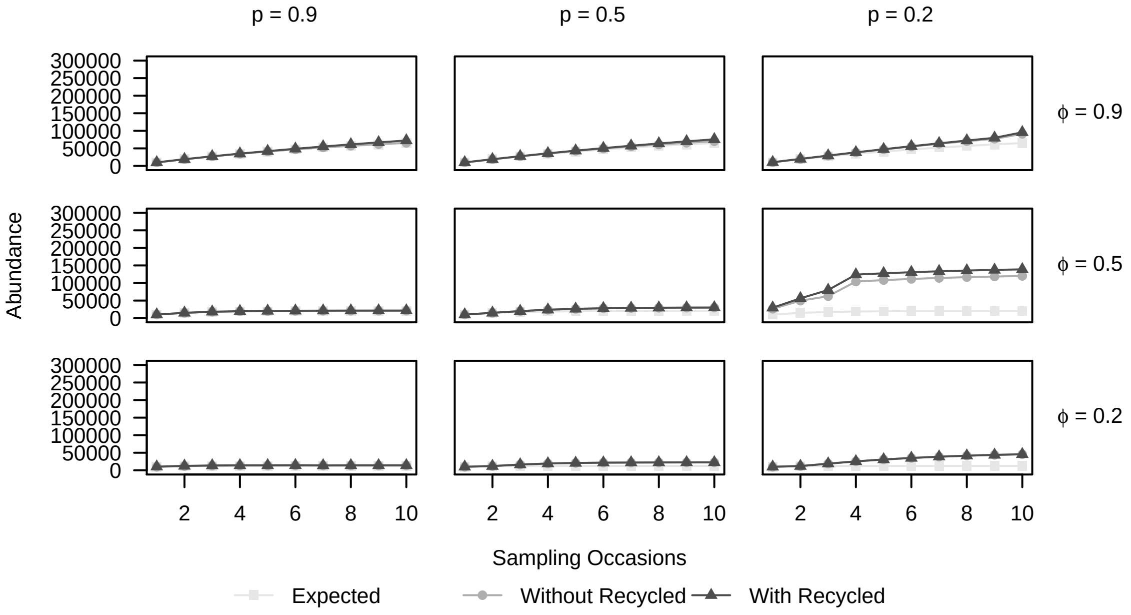

Web Figure 38: Mean abundance estimates (N_j's) for each sample time ($k = 10$) for analysis including and excluding recycled individuals with population size $N = 100000$ with $T_2 = 1$ with 10 sample times for high tag retention ($\lambda = 0.9$), varying survival probabilities ($\phi = 0.2, 0.5, 0.9$) and varying capture probabilities ($p = 0.2, 0.5, 0.9$).

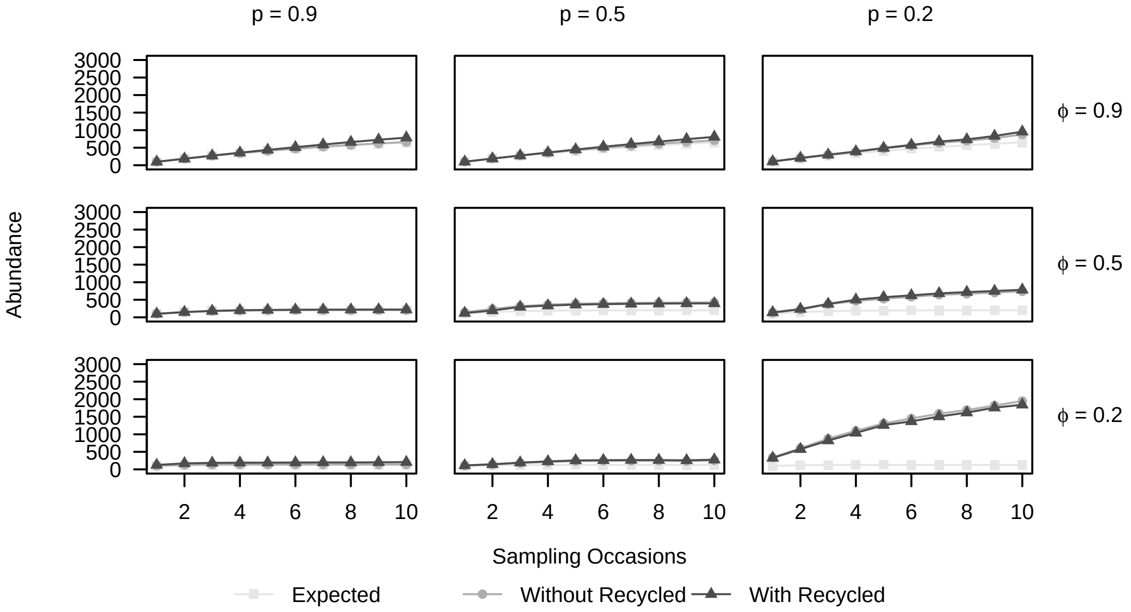

Web Figure 39: Mean abundance estimates (N_j's) for each sample time ($k = 10$) for analysis including and excluding recycled individuals with population size $N = 1000$ with $T_2 = 0.5$ with 10 sample times for high tag retention ($\lambda = 0.9$), varying survival probabilities ($\phi = 0.2, 0.5, 0.9$) and varying capture probabilities ($p = 0.2, 0.5, 0.9$).

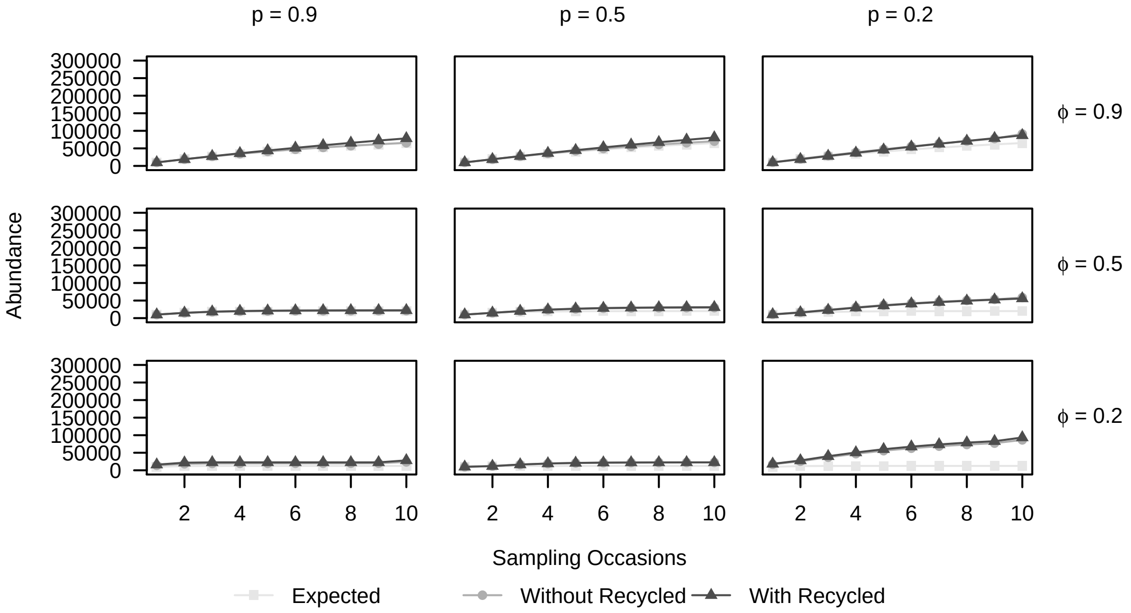

Web Figure 40: Mean abundance estimates (N_j's) for each sample time ($k = 10$) for analysis including and excluding recycled individuals with population size $N = 100000$ with $T_2 = 0.5$ with 10 sample times for high tag retention ($\lambda = 0.9$), varying survival probabilities ($\phi = 0.2, 0.5, 0.9$) and varying capture probabilities ($p = 0.2, 0.5, 0.9$).

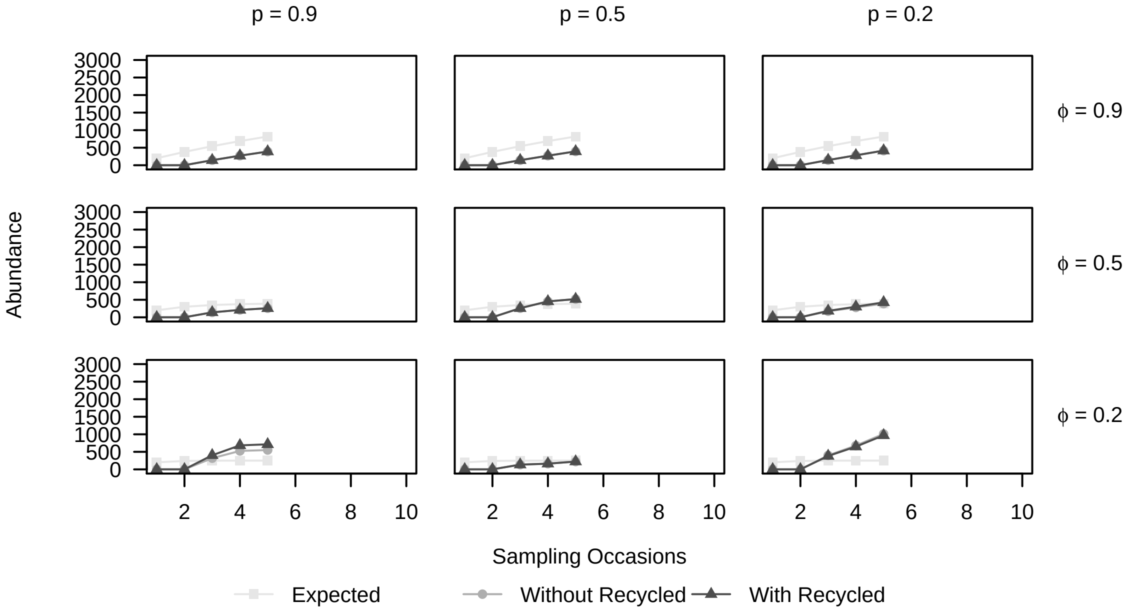

Web Figure 41: Mean abundance estimates (N_j's) for each sample time ($k = 5$) for analysis including and excluding recycled individuals with population size $N = 1000$ with $T_2 = 1$ with 5 sample times for high tag retention ($\lambda = 0.9$), varying survival probabilities ($\phi = 0.2, 0.5, 0.9$) and varying capture probabilities ($p = 0.2, 0.5, 0.9$).

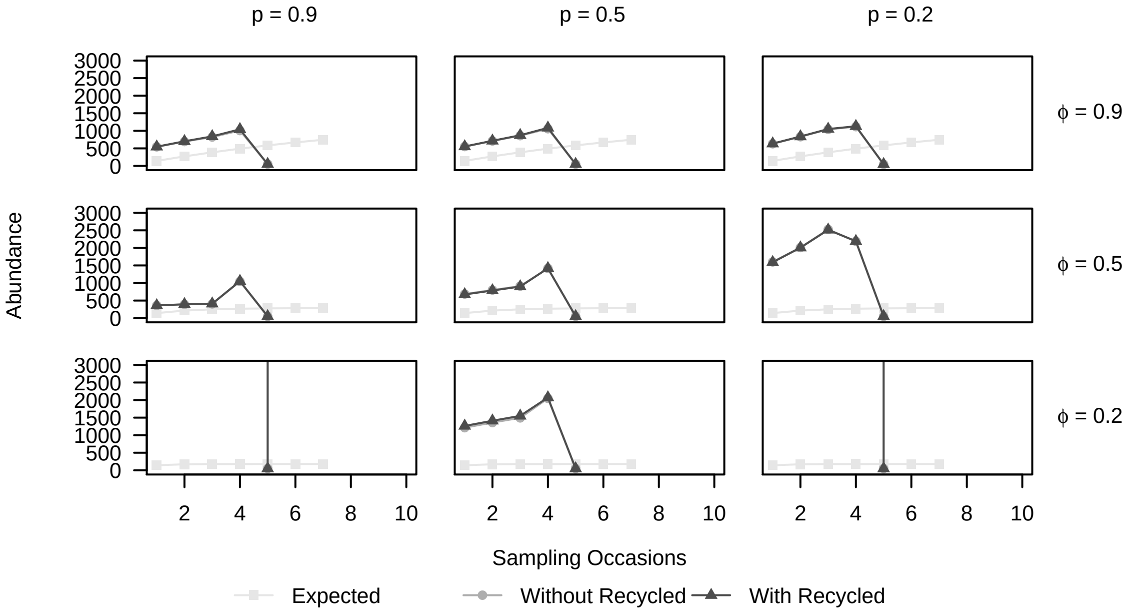

Web Figure 42: Mean abundance estimates (N_j's) for each sample time ($k = 7$) for analysis including and excluding recycled individuals with population size $N = 1000$ with $T_2 = 1$ with 5 sample times for high tag retention ($\lambda = 0.9$), varying survival probabilities ($\phi = 0.2, 0.5, 0.9$) and varying capture probabilities ($p = 0.2, 0.5, 0.9$).

Recycled Individuals

Web Table 17: Examples of the fraction of recycled individuals (number of recycled individuals captured / total number of individuals captured) at each sample time for a 10 sample-time experiment with super-population size $N = 1000$ with 100% double-tagging.

	Sampling Times									
	1	2	3	4	5	6	7	8	9	10
$\phi = 0.9, p = 0.9, \lambda = 0.2$	0.00	0.30	0.36	0.47	0.51	0.53	0.54	0.55	0.56	0.57
$\phi = 0.9, p = 0.9, \lambda = 0.5$	0.00	0.12	0.12	0.20	0.29	0.26	0.29	0.30	0.31	0.31
$\phi = 0.9, p = 0.9, \lambda = 0.9$	0.00	0.01	0.02	0.02	0.01	0.01	0.03	0.04	0.04	0.03
$\phi = 0.9, p = 0.5, \lambda = 0.2$	0.00	0.14	0.28	0.38	0.43	0.48	0.54	0.54	0.55	0.56
$\phi = 0.9, p = 0.5, \lambda = 0.5$	0.00	0.02	0.12	0.14	0.27	0.28	0.28	0.33	0.29	0.38
$\phi = 0.9, p = 0.5, \lambda = 0.9$	0.00	0.00	0.00	0.00	0.01	0.02	0.02	0.04	0.06	0.06
$\phi = 0.9, p = 0.2, \lambda = 0.2$	0.00	0.05	0.15	0.35	0.18	0.28	0.30	0.33	0.40	0.37
$\phi = 0.9, p = 0.2, \lambda = 0.5$	0.00	0.00	0.06	0.09	0.14	0.13	0.20	0.21	0.30	0.26
$\phi = 0.9, p = 0.2, \lambda = 0.9$	0.00	0.00	0.00	0.00	0.00	0.01	0.02	0.02	0.04	0.03
$\phi = 0.5, p = 0.9, \lambda = 0.2$	0.00	0.18	0.23	0.27	0.34	0.36	0.32	0.32	0.32	0.26
$\phi = 0.5, p = 0.9, \lambda = 0.5$	0.00	0.10	0.10	0.15	0.10	0.15	0.18	0.13	0.19	0.12
$\phi = 0.5, p = 0.9, \lambda = 0.9$	0.00	0.00	0.00	0.01	0.01	0.00	0.01	0.01	0.01	0.01
$\phi = 0.2, p = 0.9, \lambda = 0.2$	0.00	0.10	0.14	0.07	0.15	0.10	0.11	0.13	0.13	0.12
$\phi = 0.2, p = 0.9, \lambda = 0.5$	0.00	0.02	0.06	0.05	0.05	0.11	0.04	0.06	0.08	0.09
$\phi = 0.2, p = 0.9, \lambda = 0.9$	0.00	0.01	0.00	0.01	0.00	0.00	0.00	0.00	0.00	0.00
$\phi = 0.5, p = 0.5, \lambda = 0.5$	0.00	0.01	0.07	0.06	0.12	0.13	0.14	0.16	0.13	0.14

Web Table 18: Examples of the fraction of recycled individuals (number of recycled individuals captured / total number of individuals captured) at each sample time for a 7 sample-time experiment with super-population size $N = 1000$ with 100% double-tagging.

	Sampling Times						
	1	2	3	4	5	6	7
$\phi = 0.9, p = 0.9, \lambda = 0.2$	0.00	0.32	0.40	0.49	0.50	0.53	0.55
$\phi = 0.9, p = 0.9, \lambda = 0.5$	0.00	0.10	0.18	0.21	0.22	0.29	0.29
$\phi = 0.9, p = 0.9, \lambda = 0.9$	0.00	0.01	0.01	0.02	0.02	0.02	0.02
$\phi = 0.9, p = 0.5, \lambda = 0.2$	0.00	0.12	0.30	0.35	0.42	0.47	0.50
$\phi = 0.9, p = 0.5, \lambda = 0.5$	0.00	0.06	0.15	0.22	0.26	0.24	0.27
$\phi = 0.9, p = 0.5, \lambda = 0.9$	0.00	0.01	0.00	0.02	0.02	0.03	0.04
$\phi = 0.9, p = 0.2, \lambda = 0.2$	0.00	0.05	0.12	0.15	0.22	0.21	0.30
$\phi = 0.9, p = 0.2, \lambda = 0.5$	0.00	0.00	0.06	0.10	0.15	0.20	0.20
$\phi = 0.9, p = 0.2, \lambda = 0.9$	0.00	0.00	0.00	0.00	0.00	0.01	0.01
$\phi = 0.5, p = 0.9, \lambda = 0.2$	0.00	0.19	0.30	0.32	0.30	0.34	0.38
$\phi = 0.5, p = 0.9, \lambda = 0.5$	0.00	0.09	0.12	0.13	0.15	0.11	0.14
$\phi = 0.5, p = 0.9, \lambda = 0.9$	0.00	0.00	0.00	0.02	0.00	0.00	0.01
$\phi = 0.2, p = 0.9, \lambda = 0.2$	0.00	0.10	0.11	0.12	0.13	0.16	0.08
$\phi = 0.2, p = 0.9, \lambda = 0.5$	0.00	0.04	0.05	0.07	0.07	0.03	0.02
$\phi = 0.2, p = 0.9, \lambda = 0.2$	0.00	0.00	0.00	0.00	0.00	0.00	0.00
$\phi = 0.5, p = 0.5, \lambda = 0.5$	0.00	0.05	0.04	0.05	0.09	0.11	0.12

Web Table 19: Examples of the fraction of recycled individuals (number of recycled individuals captured / total number of individuals captured) at each sample time for a 5 sample-time experiment with super-population size $N = 1000$ with 100% double-tagging.

	Sampling Times				
	1	2	3	4	5
$\phi = 0.9, p = 0.9, \lambda = 0.2$	0.00	0.29	0.34	0.45	0.52
$\phi = 0.9, p = 0.9, \lambda = 0.5$	0.00	0.08	0.22	0.23	0.28
$\phi = 0.9, p = 0.9, \lambda = 0.9$	0.00	0.01	0.01	0.02	0.02
$\phi = 0.9, p = 0.5, \lambda = 0.2$	0.00	0.14	0.27	0.33	0.38
$\phi = 0.9, p = 0.5, \lambda = 0.5$	0.00	0.05	0.13	0.18	0.25
$\phi = 0.9, p = 0.5, \lambda = 0.9$	0.00	0.00	0.01	0.01	0.02
$\phi = 0.9, p = 0.2, \lambda = 0.2$	0.00	0.06	0.15	0.16	0.29
$\phi = 0.9, p = 0.2, \lambda = 0.5$	0.00	0.03	0.06	0.08	0.12
$\phi = 0.9, p = 0.2, \lambda = 0.9$	0.00	0.00	0.00	0.01	0.01
$\phi = 0.5, p = 0.9, \lambda = 0.2$	0.00	0.14	0.25	0.30	0.33
$\phi = 0.5, p = 0.9, \lambda = 0.5$	0.00	0.05	0.13	0.10	0.15
$\phi = 0.5, p = 0.9, \lambda = 0.9$	0.00	0.00	0.01	0.01	0.00
$\phi = 0.2, p = 0.9, \lambda = 0.2$	0.00	0.11	0.10	0.13	0.12
$\phi = 0.2, p = 0.9, \lambda = 0.5$	0.00	0.02	0.03	0.05	0.04
$\phi = 0.2, p = 0.9, \lambda = 0.9$	0.00	0.00	0.00	0.00	0.00
$\phi = 0.5, p = 0.5, \lambda = 0.5$	0.00	0.03	0.09	0.10	0.12
